# A potential implication of UDP-glucuronosyltransferase 2B10 in the detoxification of drugs used in pediatric hematopoietic stem cell transplantation setting: an in silico investigation

**DOI:** 10.1186/s12860-021-00402-5

**Published:** 2022-01-21

**Authors:** Shannon Robin, Khalil Ben Hassine, Jayaraman Muthukumaran, Simona Jurkovic Mlakar, Maja Krajinovic, Tiago Nava, Chakradhara Rao S. Uppugunduri, Marc Ansari

**Affiliations:** 1 CANSEARCH Research Platform in Pediatric Oncology and Hematology, Department of Pediatrics, Gynecology and Obstetrics, University of Geneva, Geneva, Switzerland; 2 Department of Biotechnology, School of Engineering and Technology, Sharda University, Greater Noida, 201306 India; 3 Charles-Bruneau Cancer Center, CHU Sainte-Justine Research Center, Departments of Pediatrics and Pharmacology, University of Montreal, Montreal, Quebec, Canada; 4 Division of Pediatric Oncology and Hematology, Department of Women, Child and Adolescent, University Geneva Hospitals, Geneva, Switzerland

**Keywords:** UDP-glucuronosyltransferase 2B10, Sinusoidal obstruction syndrome, Molecular dynamics, Protein-ligand docking, Homology modelling, Virtual screening

## Abstract

**Background:**

Sinusoidal occlusion syndrome (SOS) is a potentially severe complication following hematopoietic stem cell transplantation (HSCT) in pediatric patients. Treatment related risk factors such as intensity of conditioning, hepatotoxic co-medication and patient related factors such as genetic variants predispose individuals to develop SOS. The variant allele for SNP rs17146905 in UDP-glucuronosyl transferase 2B10 (*UGT2B10*) gene was correlated with the occurrence of SOS in an exome-wide association study. UGT2B10 is a phase II drug metabolizing enzyme involved in the *N*-glucuronidation of tertiary amine containing drugs.

**Methods:**

To shed light on the functionality of UGT2B10 enzyme in the metabolism of drugs used in pediatric HSCT setting, we performed in silico screening against custom based library of putative ligands. First, a list of potential substrates for in silico analysis was prepared using a systematic consensus-based strategy. The list comprised of drugs and their metabolites used in pediatric HSCT setting. The three-dimensional structure of UGT2B10 was not available from the Research Collaboratory Structural Bioinformatics - Protein Data Bank (RCSB - PDB) repository and thus we predicted the first human UGT2B10 3D model by using multiple template homology modeling with MODELLER Version 9.2 and molecular docking calculations with AutoDock Vina Version 1.2 were implemented to quantify the estimated binding affinity between selected putative substrates or ligands and UGT2B10. Finally, we performed molecular dynamics simulations using GROMACS Version 5.1.4 to confirm the potential UGT2B10 ligands prioritized after molecular docking (exhibiting negative free binding energy).

**Results:**

Four potential ligands for UGT2B10 namely acetaminophen, lorazepam, mycophenolic acid and voriconazole n-oxide intermediate were identified. Other metabolites of voriconazole satisfied the criteria of being possible ligands of UGT2B10. Except for bilirubin and 4-Hydroxy Voriconazole, all the ligands (particularly voriconazole and hydroxy voriconazole) are oriented in substrate binding site close to the co-factor UDP (mean ± SD; 0.72 ± 0.33 nm). Further in vitro screening of the putative ligands prioritized by in silico pipeline is warranted to understand the nature of the ligands either as inhibitors or substrates of UGT2B10.

**Conclusions:**

These results may indicate the clinical and pharmacological relevance UGT2B10 in pediatric HSCT setting. With this systematic computational methodology, we provide a rational-, time-, and cost-effective way to identify and prioritize the interesting putative substrates or inhibitors of UGT2B10 for further testing in in vitro experiments.

**Supplementary Information:**

The online version contains supplementary material available at 10.1186/s12860-021-00402-5.

## Background

Sinusoidal obstruction syndrome (SOS) or veno-occlusive disease is one of the complications associated with hematopoietic stem cell transplantation (HSCT) that occurs in 22–30% of pediatric HSCT patients [1]. Severe forms of SOS may lead to increased mortality rates reaching 80% [1]. The diagnosis of SOS post HSCT is based on Seattle, Baltimore or European society for blood and marrow transplantation (EBMT) criteria comprising important parameters such as increased bilirubin levels, weight gain, hepatomegaly and hemodynamical and/or ultrasound evidence of SOS [2]. In the context of HSCT in children, pre-existing liver disease, age, iron overload, and genetic variants, such as glutathione S-transferase A1 (*GSTA1*) promoter diplotypes, *CTH* genetic variant (rs1021737) [3] or *GSTM1* null genotypes are few of the important known patient related risk factors for developing post HSCT SOS [3–7]. In addition, treatment-related factors, such as high-intensity conditioning regimens comprising of two or more alkylating agents including busulfan, or the co-administration of potentially hepatotoxic prophylactic drugs such as methotrexate and cyclosporine can also contribute to the increased risk of developing SOS [4]. Exposure to these hepatotoxic injuries elicits a more permeable endothelium, permitting the entry of blood cells in the Disse space. Once a pro-inflammatory environment is created, cellular debris accumulate in hepatic blood vessels. This cascade of events leads to the reduction of venous outflow and finally obstruction of centrilobular veins [8, 9]. Patients’ individual germ-line genetic polymorphisms can increase the risk of treatment related complications [4, 5]. In search of predictive genetic variants for increased risk of post HSCT SOS development in children, our group recently conducted and exome-wide association study (EWAS) that identified three variants rs17146905, rs16931326, rs2289971 in *UGT2B10*, *BHLHE22,* and *KIAA1715* genes, respectively [10]. The three independent associations from EWAS were validated in a separate cohort after adjustment with the known risk factors of SOS [10]. Multivariate analysis performed in a replication cohort confirmed the important implication of 2 of those SNPs in the occurrence of SOS: *KIAA1715* single nucleotide polymorphism (SNP) (rs16931326), coding for lunapark protein, involved in the formation of the endoplasmic reticulum and a potential drug metabolizing enzyme gene *UGT2B10* SNP (rs17146905) [10]. The latter is located in 3′-untranslated regions (3′-UTR) of *UGT2B10 *which might affect its gene expression by regulating the messenger RNA (mRNA)-related processes, such as localization and stability of mRNA, or by directly modulating protein conformation inducing a potential change in the enzyme activity [11]. Interestingly, UGT2B10 expression and activity were demonstrated to be regulated by the microRNA (miRNA) miR-216b-5p, which binds to specific target miRNA recognition element located in the 3′-UTR part [12], comprising SNP rs139538767, which is known to alter the *UGT2B10* gene regulation. However, the relationship between this SNP and the altered protein function is yet to be elucidated. It is interesting to note that the *UGT2B10* SNP (rs17146905) is in strong linkage disequilibrium with the non-synonymous SNP rs61750900 located in exonic region (R^2^ ≥ 0.8), in American and European populations (Data not shown). The latter genetic variant might also have an influence on UGT2B10 metabolizing capacity. Furthermore, the role of UGT2B10 in the metabolism of drugs used in the HSCT setting is not clearly known.

UGT2B10 belongs to a group of UDP-glucuronosyltransferases (UGTs, EC 2.4.1.17), which are phase II conjugating drug metabolizing enzymes catalyzing the glucuronidation of endogenous compounds and xenobiotics [13]. The reaction involves the conjugation of the glucuronic acid group of UDP-glucuronic acid (UDPGlcA) to a nucleophilic acceptor (oxygen, nitrogen, or sulfur) that could be a rate limiting step in clearance of drugs/xenobiotics. Based on amino acid sequence similarities and identities, UGTs in humans are divided into four families (1,2,3 and 8), but glucuronidation reactions are catalyzed by UGT1 and UGT2 families comprising a total of 19 isoforms [14]. The UGT2 family is divided into two subgroups: UGT2A and UGT2B, comprising 3 and 7 isoforms, respectively. UGTs are ubiquitous proteins, principally expressed in the liver, the gastrointestinal tract, and the kidneys [15]. The crucial implication of genetic variants altering UGTs’ function and consequently in the metabolism of drugs including anti-cancer therapies is well-known [16].

UGT2B10 is one of the liver specific UGT isoforms that has been shown glucuronidation activity similar to that of several other isoforms [17]. However, protein quantification experiments using LC-MS/MS from limited number of human (adults) liver microsomal preparations indicated relatively lower abundance of UGT2B10 compared to other UGTs [18]. Similar to UGT1A3 and UGT1A4, UGT2B10 catalyzes *N*-glucuronidation of endogenous and exogenous compounds, with a higher affinity towards tertiary aliphatic and heterocyclic amines [19]. Interestingly, UGT1A4 and UGT2B10 have common substrates such as antipsychotic and antidepressant drugs; for e.g. cyclobenzaprine, mirtazapine or clozapine [20]. As the UGT2B10 isoform is not known to catalyze *O*-glucuronidation, it was often considered as an orphan enzyme after the unsuccessful screening of large panels of substrates. However, in the last decade, after the demonstration of the primary role of UGT2B10 in the glucuronidation of tobacco-related nitrosamines [21], many other substrates were discovered. Examples of such ligands include antidepressants, antifungals, and chemotherapeutics; e.g. fluconazole, midazolam or imatinib [19, 22] as well as arachidonic and linoleic acid metabolites [23]. An exhaustive list of UGT2B10 drug ligands can be found in Additional file 1**.**

Other members of the UGT enzymatic family (UGT1A1, 1A3, 1A6, 1A7, and 1A10) were demonstrated to be involved in the metabolism of drugs such as acetaminophen, lorazepam or deferasirox used in pediatric HSCT setting [24]. However, to the best of our knowledge, the evidence is lacking for the role of UGT2B10 in the metabolism of potential hepatotoxic drugs and/or their metabolites used in the pediatric HSCT setting. Regarding its role in the detoxification of a large panel of drugs, the altered activity of UGT2B10 due to drug-drug interactions or genetic variants may modulate the exposure to hepatotoxic amine- compounds used in HSCT setting administered along with a busulfan, known for its hepatotoxic effect. Besides, some compounds may also interact with UGT2B10 as enzyme inhibitors or activators and differentially regulate the *N-*glucuronidation of toxic compounds. For instance, such interactions were already demonstrated for fluconazole, which is an inhibitor of UGT2B10 [25].

Hence, we aimed to explore the role of UGT2B10 in the detoxification of commonly used medication in pediatric HSCT setting. We attempted to establish an in silico workflow for screening of the ligands using a homology modelled UGT2B10 protein. The potential ligands for further in vitro screening were identified based on results obtained from extensive molecular docking and molecular dynamics simulations.

## Results

### Generation of the three-dimensional model of UGT2B10

**Fig. 1:**
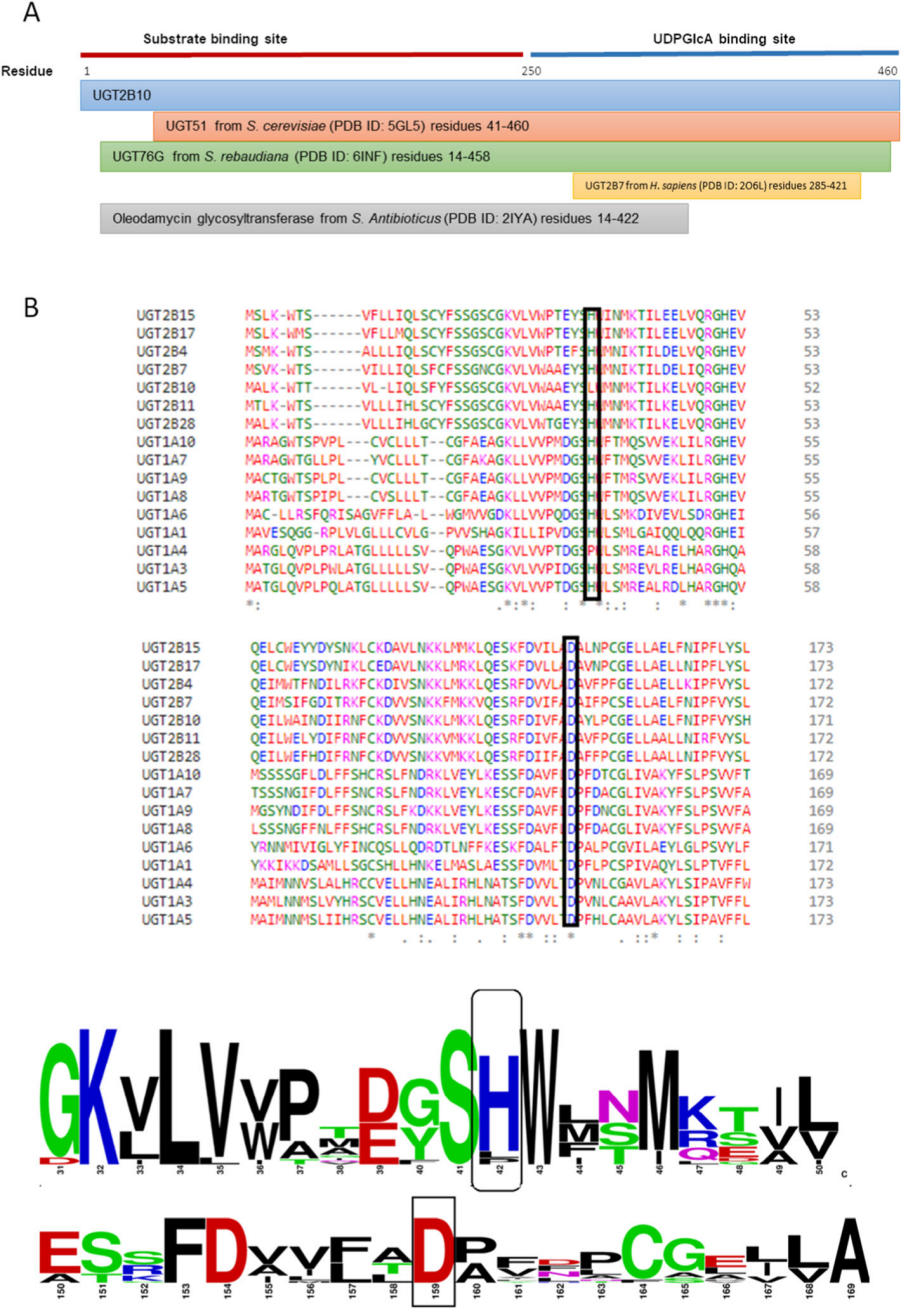
(**A**): Multiple sequence alignment of the template structures for homology modeling and the query sequence of UGT2B10. (**B**): Multiple sequence alignment of N-terminal region (substrate binding site) of various members of UGT family. Putative catalytic bases are highlighted in black box

The UGT2B10 protein sequence comprised of 460 amino-acid residues without the signal peptide, transmembrane region and 8 residues surrounding transmembrane region. 250 potential templates or structural neighbors, based on Hidden-Markov model (HMM) profile similarities, were retrieved from HHpred [26, 27] and filtered to select four suitable structural templates. Human UGT2B10 model was built with the following related protein structures or templates; UGT51 from *S. cerevisiae* (PDB ID: 5GL5), UGT76G from *S. rebaudiana* (PDB ID: 6INF), UGT2B7 from *H. sapiens* (PDB ID: 2O6L), and oleodamycin glycosyltransferase from *S. antibioticus* (PDB ID: 2IYA) (Table 1) were used to build the multi-sequence alignment (MSA). The multiple sequence alignment covering UGT2B10 query sequence of 460 residues is given in Figure 1. Sequence alignment with the members of UGT family revealed putative catalytic residue i.e. D150 which is conserved and another residue H34 which is not conserved similar to that of UGT1A4.

**Table 1:** Characteristics of the four structural templates used to build the homology model of UGT2B10

Protein name	Organism	PDB ID	InterPro-Protein Family	Resolu-tion [Å]	Method	Sequence identity with the templates (%)	Secondary structure similarity with the templates (%)	Query coverage with the templates (%)
Sterol 3-beta-glucosyltransferase ATG26, UGT51	*Saccharomyces cerevisiae* S288c	5GL5	Glucosyl transferase family	1.9	X-ray	20.7	40.7	38
UGT76G	*Stevia rebaudiana*	6INF	UDP Glycosyl transferase family	1.7	X-ray	30.3	33.1	26
UGT2B7	*Homo sapiens*	2O6L	UDP glucuronosyl ransferase family	1.8	X-ray	91	22.4	33
Oleodamycin glycosyltransferase	*Streptomyces antibioticus*	2IYA	UDP Glycosyl transferase family	1.7	X-ray	28.5	34.1	19

**Fig. 2:**
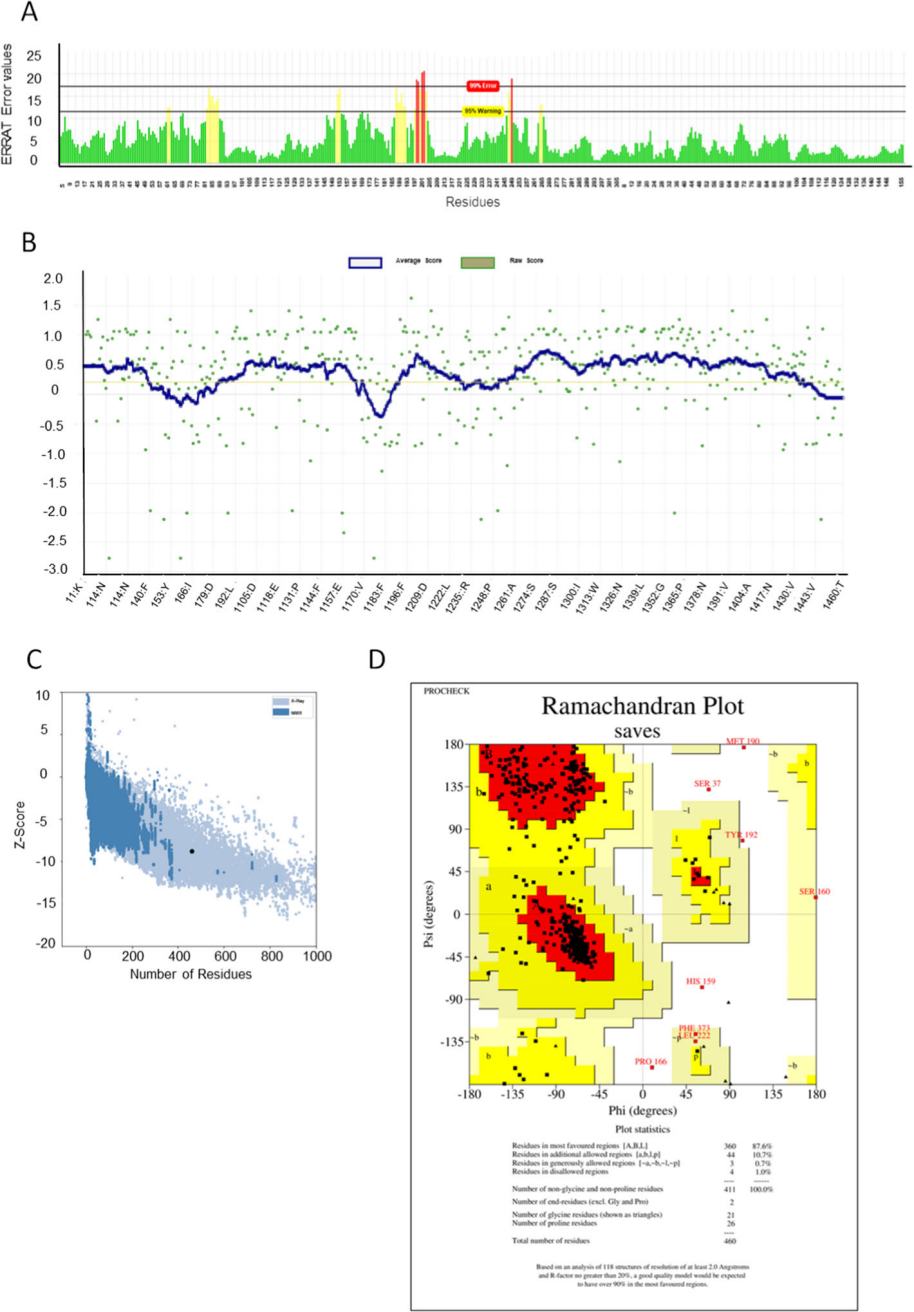
Structure validation results of homology model of UGT2B10. **A**) ERRAT graph. The ERRAT score was 93.3%, X-axis represents theamino acid residues of the protein model and Y-axis represents the error value **B**) Verify3D profile. The average score was 74.8% **C**) ProSA (Z-score) plot. The model was situated on the region of structures obtained by X-ray, and with a score of − 8.78. **D**) Ramachandran plot. 87.6% of residues were situated on the most favored region of the graph

To obtain the final human UGT2B10 homology model, six steps of structure refinement were performed, three with GalaxyRefine [28] followed by three steps with Yasara minimization server [29] (Additional file 2). The reason for using two minimization programs is to identify the optimized and reliable conformation of UGT2B10 for further molecular docking and MD simulation with putative ligands. The UGT2B10 model built using multiple templates had an ERRAT (overall quality factor) [30] score of 93.3% (Figure 2A), a Verify3D (compatibility of an atomic model) [31, 32] score of 74.8% (Figure 2B), and a ProSA (overall stereochemical quality of the protein structure) [33] score of − 8.78 (Figure 2C). 92.8% of the residues are situated in the favored region of the Ramachandran plot, whereas 1.7% were situated in the outlier region (Figure 2D). The overall structure comprised 460 amino acids, the secondary structure composed of 35% of alpha helix, and three parallel beta sheets (Additional file 3). This protein structure was further used in in silico virtual screening of selected ligands.

**Fig. 3:**
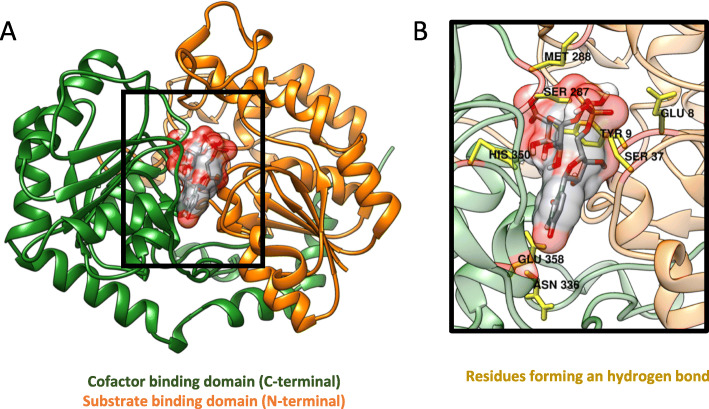
**A** Multi-template-based homology model of human UGT2B10 obtained with MODELLER and bound with cofactor UDPGlcA. **B** Close-up representation of the cofactor binding site

The co-factor (UDPGlcA) binding site was predicted by superposition of the UGT2B10 model with the structural templates used for homology or comparative protein modelling. The cofactor binding box had a size of (x,y,z) = (15, 17, 22.5) and center coordinates of (x,y,z) = (− 70, 8, 3). The grid box of substrate-binding site had a size of (x,y,z) = (22, 20, 25) and center coordinates of (x,y,z) = (− 70, 7, 15) (Additional file 1) UDPGlcA was successfully bound to the human UGT2B10 model (Fig. 3A), with an estimated free energy of binding (*ΔG*) of − 7.0 kcal/mol (Kd = 7.3 μM). Hydrogen bonds are formed between the UDPGlcA and residues of UGT2B10 namely Glu8, Tyr9, Ser37, Ser287, Met288, Asn336, His350, and Glu358 (Figure 3B).

### Selection of putative UGT2B10 ligands

**Fig. 4:**
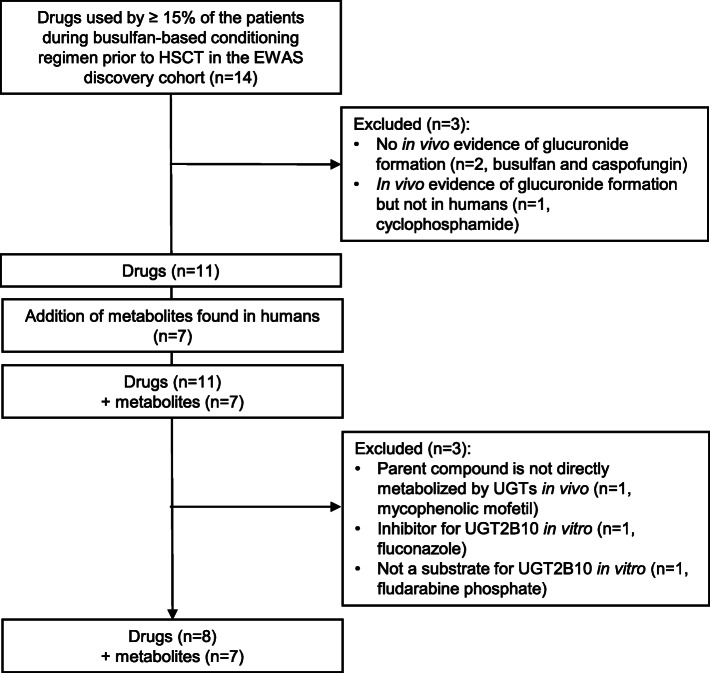
Pipeline of selection of putative UGT2B10 ligands for molecular docking. Drugs and metabolites were selected based on clinical, chemical, and biological criteria. Molecules were excluded from the selection pipeline as in vitro or in vivo evidence showed that the molecule were not undergoing glucuronidation

Eight drugs and seven metabolites of drugs used in HSCT setting are identified as putative UGT2B10 substrates using a consensus-based approach (Figure 4, Table 2). The chemical structures of the molecules are presented in the Additional file 5. Three ligands (cyclosporine A, methotrexate, and posaconazole) are tertiary amines, the favored substrates of UGT2B10, and two are described as forming *N*-glucuronide conjugates. Four ligands have reported liver toxicity. The missing information is due to lack of clinical or in vitro data about the compound metabolism (Table 2).

**Table 2:** Refined list of putative UGT2B10 ligands to be tested with docking predictions simulations

	Pharmacology	Hepatotoxicity			Glucuronidation			
Compound	Indication in HSCT setting / metabolite of drug	LiverTox database category*	liver toxicity data from curated source (Medscape)	Reported risk factor for SOS [4]	UGTs involved	Type of glucuronidation	Amine group presence	Sources
**4-hydroxyvoriconazole**	Voriconazole metabolite	N/A	N/A	N/A	UGT1A4 (hypothetical)	O-glucuronidation	Secondary amine	[34, 35]
**Cyclosporine A**	GvHD prophylaxis	C	Yes	Yes	UGT2B7	N/A	Tertiary amine	[36]
**Dihydroxy voriconazole**	Voriconazole metabolite	N/A	N/A	N/A	UGT1A4 (hypothetical)	O-glucuronidation	Secondary amine	[34, 35]
**Hydroxymethyl voriconazole**	Voriconazole metabolite	N/A	N/A	N/A	UGT1A4 (hypothetical)	O-glucuronidation	Secondary amine	[34, 35]
**Lorazepam**	Seizure prophylaxis	E	Yes	No	S-lorazepam: UGT2B4 UGT2B7 UGT2B15 R-lorazepam: UGT2B4 UGT2B7 UGT2B15 UGT1A7 UGT1A10	O-glucuronidation	Secondary amine	[37]
**Methotrexate**	GvHD prophylaxis	A	Yes	Yes	UGT1A6	N-glucuronidation	Tertiary amine	[38]
**Methylprednisolone**	GvHD prophylaxis	A	Yes	Yes	N/A	O-glucuronidation	Non-amine	[39, 40]
**Mycophenolic acid**	The active metabolite of mycophenolate mofetil GvHD prophylaxis	D	Yes	No	UGT1A8 UGT1A9 UGT1A1 (minor) UGT1A7 (minor) UGT1A10 (minor) UGT2B7 (minor)	O-glucuronidation	Non-amine	[41]
**Acetaminophen**	Analgesic, Antipyretic	A	Yes	No	UGT1A1 UGT1A6 UGT1A9 UGT2B15	O-glucuronidation	Secondary amine	[42]
**Posaconazole**	Antifungal	E	Yes	No	UGT1A4	O-or N-glucuronidation	Tertiary amine	[43]
**UDCA-G1**	Ursodeoxycholic acid metabolite	N/A	N/A	N/A	UGT1A3 UGT1A4 UGT2B7 UGT2B7	N/A	Non-amine	[44]
**UDCA-G2**	Ursodeoxycholic acid metabolite	N/A	N/A	N/A	UGT1A4 UGT2B7	N/A	Non-amine	[44]
**Ursodeoxycholic acid**	SOS prophylaxis	D	Yes?	Used as SOS prophylaxis	UGT1A3 UGT1A8 UGT1A9 UGT2B7 UGT2B17	O-glucuronidation	Non-amine	[44]
**Voriconazole**	Antifungal	B	Yes	No	UGT1A4	O-glucuronidation	Secondary amine	[34, 35]
**Voriconazole N-oxide**	Voriconazole metabolite	N/A	N/A	N/A	N/A	O-glucuronidation	Secondary amine	[34, 35]
**Voriconazole N-oxide imtermediate UK-215,364** [35]	Voriconazole metabolite	N/A	N/A	N/A	N/A	O-glucuronidation	Secondary amine	[34, 35]

*From LiverTox classification of the molecules regarding the potential to induce liver injury: A: Well known cause, B: Known or highly likely, C: Probable, D: Possible, E: not believed or unlikely [45]. GvHD: Graft versus host disease; HSCT: hematopoietic stem cell transplantation;N/A: not available data; SOS: sinusoidal occlusion syndrome; UDCA-G1 and UDCA-G2: ursodeoxycholic acid glucuronide conjugate 1 and 2 [44]. The structure of the molecules are presented in the additional file

### Molecular docking analysis

**Fig. 5:**
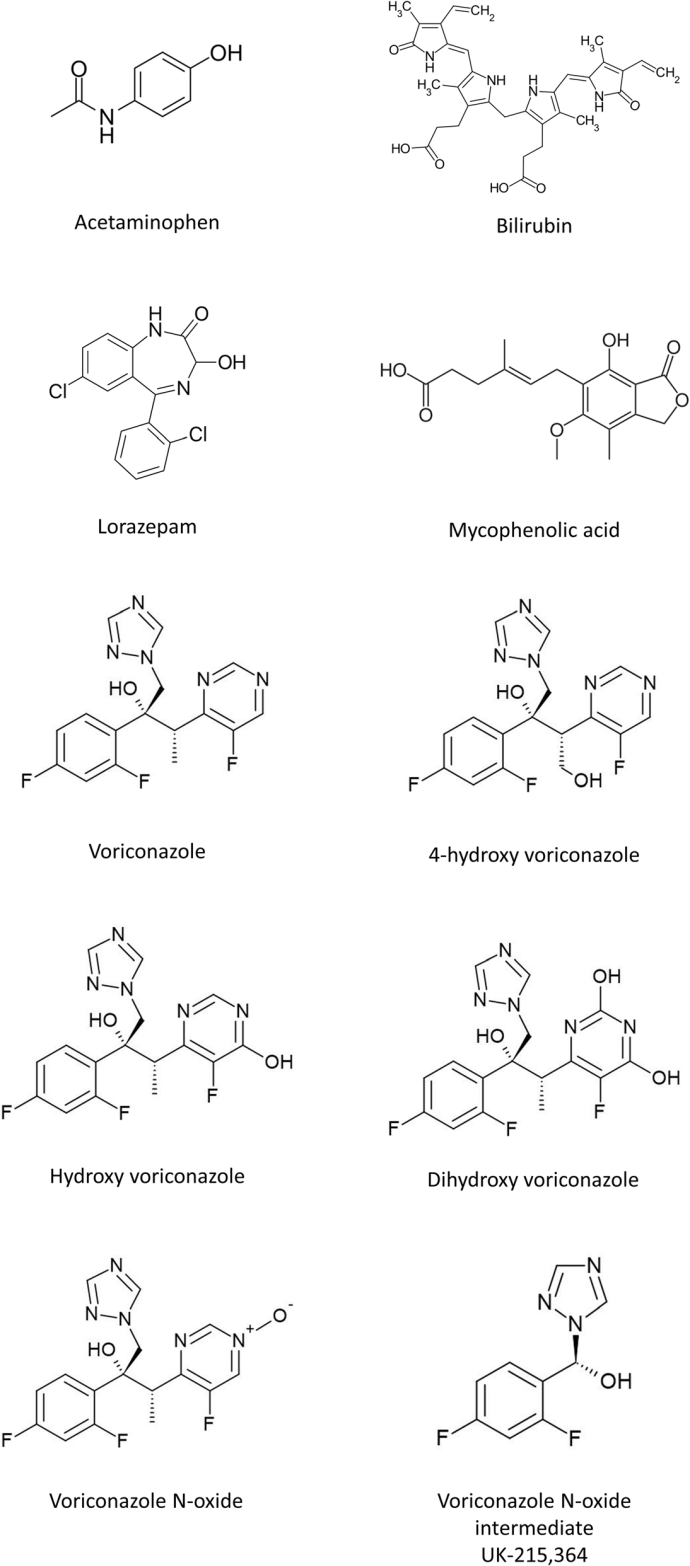
Chemical structure of the putative UGT2B10 ligands selected with molecular docking analyses in AutoDock Vina [47]. Structures were obtained with SMILES explorer [48]

The positive control amitriptyline (AMT) had *ΔG* of − 1.9 kcal/mol, whereas itraconazole (ITZ, chosen negative control based on the available literature [25]) had 19 kcal/mol, indicating that the ligand binding and interaction does not occur spontaneously. These results indicated that our predicted model of UGT2B10 is reliable, therefore has been implemented for the selection of putative UGT2B10 ligands. A negative *ΔG* value indicates that the reaction is spontaneous, thus that the conformation between the ligand and the enzyme is favorable. The more negative the results, the more affinity between the ligand and protein. Recently, after we built the homology model of UGT2B10, three-dimensional structure of UGT2B10 was predicted using an Artificial Intelligence approach by deepmind team and was retrieved from the AlphaFold structure database [46]. This model exhibited better structure validation results (Additional file 2) and was utilized for the comparison with our model using molecular docking with experimentally proven substrates / inhibitors of UGT2B10 [25] (chemical structures of the selected molecules are given in Additional file 5). The comparison of the UGT2B10 structure between our model and Alpha Fold model showed a RMSD of 2.98 Å **(**Additional file 4C**)**. Although, the RMSD value is slightly high, the overall architecture of two in silico models are nearly similar, interestingly, the position and orientation of UDP is also consistent in both models indicating that our model is reliable, and it does not have any geometry or other stereochemical errors as demonstrated using various structure validation tools. However, the secondary structural composition of both the models are slightly different from each other (Additional file 2). Molecular docking with AlphaFold model resulted in negative free binding energies for all the molecules including negative controls (Additional file 6) Whereas our model resulted in negative free binding energies for 4 out of 6 negative controls. No linear correlation between the predicted free binding energy and experimental IC_50_ results was observed for our model or the AlphaFold model (Additional file 7). The results clearly indicate that our model has relatively increased sensitivity to detect negative controls compared to that of AlphaFold model. Following molecular docking of ligands with our model, 10 molecules were selected for further MD simulation analyses including the positive control AMT, negative control ITZ, and the complex formed only by the protein and the cofactor UDPGlcA (Table 3, Fig. 5). Moreover, the reason for differences in docking results obtained from two different models may be explained by the differences in the secondary structural composition (Additional file 2).

**Table 3:** Estimated binding free energy and dissociation constant between putative substrates and human UGT2B10

Model	Substrate	Ligand	*ΔG* [Kcal/mol ± SD]	Kd [mM]
**UGT2B10 with UDPGlcA**	**Controls**	**Amitriptyline**	**−1.9 ± 0.2**	**39.0**
		**Itraconazole**	19.0 ± 0.5	1.1 × 10^17^
	**Putative ligands**	**4-hydroxy voriconazole**	**−1.0 ± 0.0**	**184.7**
		**Acetaminophen**	**−5.5 ± 0.0**	**0.1**
		Cyclosporine A	154.9 ± 2.9	1.8 × 10^118^
		Bilirubine	6.9 **± 0.0**	1.2 × 10^15^
		Dihydroxy voriconazole	−0.6 ± 0.0	363.0
		**Hydroxy voriconazole**	**−1.2 ± 0.1**	**125.0**
		**Lorazepam**	**−2.6 ± 0.0**	**12.4**
		Methotrexate	−0.5 ± 0.5	567.3
		Methylprednisolone	5.2 ± 0.1	6.2 × 10^6^
		**Mycophenolic acid**	**−5.1 ± 0.1**	**0.2**
		Posaconazole	17.6 ± 0.3	8.8 × 10^15^
		UDCA-G1	2.2 ± 0.1	4.4 × 10^4^
		UDCA-G2	1.2 ± 0.1	8053.6
		Ursodeoxycholic acid	2.2 ± 0.1	4.4 × 10^4^
		**Voriconazole**	**−1.0 ± 0.1**	**197.8**
		**Voriconazole N-oxide**	**−2.3 ± 0.1**	**2.1 × 10**^**4**^
		**Voriconazole N-oxide intermediate UK-215,364** [35]	**−6.4 ± 0.1**	**0.02**

Results are presented as the mean ± SD of three different replicates. Kd: dissociation constant; UDCA-G1 and UDCA-G2: ursodeoxycholic acid glucuronide conjugate 1 and 2 [44]; UDPGlcA: UDP-glucuronic acid. Molecules with ΔG of < −0.1 and with an SD of ≤0.1 Kcal/mol were selected for further for MD simulations (methotrexate was not selected as it has an SD 0.5). SD is calculated from 8 docking poses or models (default option). The ligand binding pose was selected for further analyses is the pose with the lowest free binding energy (Kcal/mol). Bilirubin was selected for further molecular docking simulations as an endogenous negative control to compare our results with other putative ligands.

**Fig. 6:**
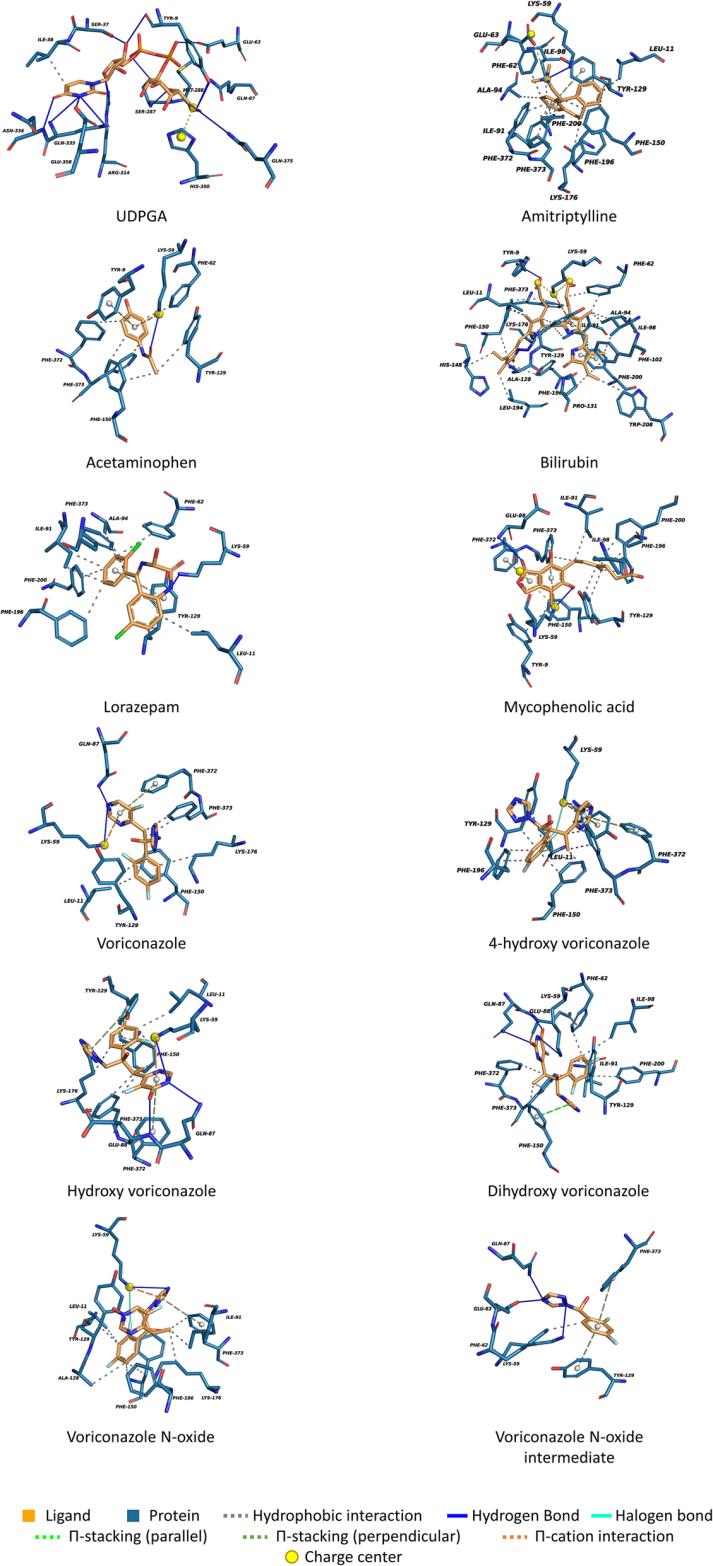
Structural representation of the residues interacting with the putative ligands

The putative compound with the highest predicted affinity toward UGT2B10 is the voriconazole metabolite voriconazole *N*-oxide intermediate UK-215,364 (VCZ-N-O intermediate), followed by acetaminophen (APAP), mycophenolic acid (MPA), and lorazepam (LOR) (Table 3, Figure 5). The residues interacting with AMT were consistent with the ones involved in the binding with other putative UGT2B10 substrates, APAP, LOR, MPA and VCZ-N-O intermediate (Table 4, Figure 6). However, the predicted hydrogen-bonds between protein and various ligands were different. Hydrogen bond with residue Lys59 is predicted to be formed for all the ligands investigated except for BIL. Hydrogen bond interaction and other molecular interactions of ligands with protein residues are given in Table 4.

**Table 4:** Important residues interacting with the putative UGT2B10 substrates obtained after docking calculations with AutoDock Vina. These results were obtained with Protein Ligand Interaction Profiler (PLIP) [49]

UDPGlcA	**Hydrophobic interactions**		
	Residue	Distance	Ligand atom
	38A ILE	3.34	3
	**Hydrogen bonds**		
	Residue	Distance H-A	Donor – acceptor atom
	9A TYR	2.79	36 [O3] - 128 [O2]
	9A TYR	2.45	136 [O3] - 31 [O.co2]
	37A SER	2.9	404 [O3] - 36 [O3]
	63A GLU	3.17	22 [O3] - 654 [O.co2]
	87A GLN	3.13	904 [Nam] - 31 [O.co2]
	287A SER	2.11	2894 [O3] - 19 [O3]
	287A SER	2.25	2889 [Nam] - 29 [O3]
	288A MET	3.12	2897 [Nam] - 29 [O3]
	314A ARG	2.23	3154 [Ng+] - 15 [O3]
	314A ARG	3.04	3155 [Ng+] - 15 [O3]
	335A GLN	3.06	3362 [Nam] - 8 [Nam]
	335A GLN	2.99	3369 [Nam] - 8 [Nam]
	336A ASN	2.06	3374 [Nam] - 10 [O2]
	358A GLU	1.95	8 [Nam] - 3586 [O-]
	375A GLN	3.48	3737 [Nam] - 31 [O.co2]
Amitriptyline	**Hydrophobic interactions**		
	Residue	Distance	Ligand atom
	11A LEU	2.8	11
	62A PHE	3.12	19
	91A ILE	3.76	18
	94A ALA	3.78	19
	98A ILE	3.9	18
	129A TYR	3.01	11
	129A TYR	3.45	10
	129A TYR	3.19	8
	150A PHE	2.91	11
	150A PHE	2.88	12
	150A PHE	3.41	5
	176A LYS	3.33	14
	196A PHE	3.48	15
	200A PHE	3.37	18
	372A PHE	3.79	5
	373A PHE	3.57	5
	373A PHE	2.71	6
	**Hydrogen bonds**		
	Residue	Distance H-A	Donor – acceptor atom
	59A LYS	2.41	586 [N3+] - 2 [N3]
Itraconazole	**Hydrophobic interactions**		
	Residue	Distance	Ligand atom
	9A TYR	3.09	46
	91A ILE	3.58	30
	94A ALA	2.87	30
	95A ILE	3.33	17
	98A ILE	3.92	17
	102A PHE	3.41	18
	129A TYR	2.98	26
	131A PRO	2	14
	135A LEU	3.54	4
	150A PHE	2.38	48
	198A PHE	2.69	6
	200A PHE	3.94	15
	200A PHE	3.59	17
	200A PHE	3.89	30
	208A TRP	4	4
	208A TRP	3.32	18
	212A TYR	3.29	4
	372A PHE	3.07	44
	373A PHE	3.49	27
	373A PHE	2.67	49
	373A PHE	3.43	29
	**Hydrogen bonds**		
	Residue	Distance H-A	Donor – acceptor atom
	59A LYS	2.47	614 [N3+] - 41 [Nar]
	227A ARG	3.22	2322 [Ng+] - 9 [O2]
	**Halogen bonds**		
	Residue	Distance	Donor – acceptor atom
	87A GLN	2.68	2 [Cl] - 907 [Nam]
Acetaminophen (APAP)	**Hydrophobic interactions**		
	Residue	Distance	Ligand atom
	62A PHE	3.35	9
	129A TYR	3.67	1
	150A PHE	3.43	1
	150A PHE	3.39	6
	372A PHE	3.68	7
	373A PHE	3.24	10
	**Hydrogen bonds**		
	Residue	Distance H-A	Donor – acceptor atom
	59A LYS	2.2	578 [N3+] - 3 [O2]
Bilirubine	**Hydrophobic interactions**		
	Residue	Distance	Ligand atom
	11A LEU	3.07	28
	62A PHE	2.33	1
	62A PHE	3.03	8
	91A ILE	3.73	12
	94A ALA	3.04	1
	98A ILE	3.03	43
	98A ILE	2.63	1
	102A PHE	3.68	41
	128A ALA	2.07	26
	129A TYR	3.58	34
	129A TYR	3.2	14
	131A PRO	2.55	41
	148A HIS	3.73	27
	150A PHE	3.58	20
	150A PHE	2.72	28
	150A PHE	3.47	30
	176A LYS	2.98	12
	194A LEU	3.35	25
	196A PHE	3.97	21
	200A PHE	3.3	34
	208A TRP	3.27	42
	373A PHE	3.4	30
	373A PHE	2.51	7
	**Hydrogen bonds**		
	Residue	Distance H-A	Donor – acceptor atom
	9A TYR	2.56	33 [O.co2] - 139 [O3]
	176A LYS	2.29	1778 [N3+] - 17 [Npl]
Lorazepam	**Hydrophobic interactions**		
	Residue	Distance	Ligand atom
	11A LEU	2.93	9
	62A PHE	3.82	14
	91A ILE	3.77	16
	91A ILE	3.42	15
	94A ALA	3.97	14
	129A TYR	2.8	11
	129A TYR	3.27	3
	196A PHE	3.59	16
	200A PHE	2.93	15
	373A PHE	3.37	12
	**Hydrogen bonds**		
	Residue	Distance H-A	Donor – acceptor atom
	59A LYS	1.67	588 [N3+] - 1 [Nam]
Mycophenolic acid	**Hydrophobic interactions**		
	Residue	Distance	Ligand atom
	9A TYR	3.87	1
	91A ILE	3.88	14
	98A ILE	2.94	18
	129A TYR	3.69	17
	129A TYR	3.21	16
	150A PHE	3.16	1
	196A PHE	3.11	17
	200A PHE	3.41	18
	200A PHE	3.42	16
	373A PHE	3.2	14
	**Hydrogen bonds**		
	Residue	Distance H-A	Donor – acceptor atom
	59A LYS	3.52	590 [N3+] - 12 [O3]
	88A GLU	2.57	888 [Nam] - 7 [O2]
Voriconazole	**Hydrophobic interactions**		
	Residue	Distance	Ligand atom
	11A LEU	3.56	18
	129A TYR	3.13	16
	150A PHE	2.97	18
	150A PHE	3.76	20
	150A PHE	2.61	8
	176A LYS	3.82	21
	373A PHE	3.25	8
	373A PHE	2.72	7
	**Hydrogen bonds**		
	Residue	Distance H-A	Donor – acceptor atom
	59A LYS	2.95	591 [N3+] - 2 [Nar]
	87A GLN	2.77	884 [Nam] - 2 [Nar]
Hydroxy voriconazole	**Hydrophobic interactions**		
	Residue	Distance	Ligand atom
	11A LEU	3.58	22
	129A TYR	3.16	18
	150A PHE	3	22
	150A PHE	3.71	20
	150A PHE	2.62	10
	176A LYS	3.79	19
	373A PHE	3.36	10
	373A PHE	2.67	9
	**Hydrogen bonds**		
	Residue	Distance H-A	Donor – acceptor atom
	59A LYS	2.96	593 [N3+] - 14 [Nar]
	87A GLN	2.83	886 [Nam] - 14 [Nar]
	88A GLU	2.45	891 [Nam] - 16 [O3]
dihydroxy voriconazole	**Hydrophobic interactions**		
	Residue	Distance	Ligand atom
	62A PHE	2.83	21
	91A ILE	3.35	23
	98A ILE	3.65	21
	129A TYR	2.92	20
	150A PHE	2.93	10
	200A PHE	3.79	23
	372A PHE	3.65	9
	373A PHE	3.17	19
	373A PHE	2.95	10
	**Hydrogen bonds**		
	Residue	Distance H-A	Donor – acceptor atom
	59A LYS	2.92	595 [N3+] - 15 [N2]
	87A GLN	3.12	888 [Nam] - 14 [O2]
	88A GLU	2.28	893 [Nam] - 17 [O2]
4-Hydroxyvoriconazole	**Hydrophobic interactions**		
	Residue	Distance	Ligand atom
	11A LEU	3.74	12
	129A TYR	3.01	12
	129A TYR	3.11	10
	150A PHE	2.84	12
	150A PHE	2.99	1
	196A PHE	3.47	14
	196A PHE	3.39	15
	373A PHE	3.02	1
	373A PHE	2.74	2
	**Hydrogen bonds**		
	Residue	Distance H-A	Donor – acceptor atom
	59A LYS	2.11	593 [N3+] - 6 [Nar]
	**Halogen bonds**		
	Residue	Distance	Donor atom
	59A LYS	3.15	25 [F] - 593 [N3+]
Voriconazole-N-oxide	**Hydrophobic interactions**		
	Residue	Distance	Ligand atom
	11A LEU	3.63	18
	91A ILE	2.98	8
	128A ALA	3.92	20
	129A TYR	3.05	18
	150A PHE	3.78	20
	176A LYS	3.71	8
	196A PHE	3.57	20
	373A PHE	3	8
	**Hydrogen bonds**		
	Residue	Distance H-A	Donor – acceptor atom
	59A LYS	2.91	592 [N3+] - 5 [Nar]
	**Halogen bonds**		
	Residue	Distance	Donor – acceptor atom
	59A LYS	3.55	25 [F] - 592 [N3+]
Voriconazole-N-oxide intermediate UK 215,364 [35]	**Hydrophobic interactions**		
	Residue	Distance	Ligand atom
	62A PHE	3.48	4
	**Hydrogen bonds**		
	Residue	Residue	Residue
	59A LYS	2.42	583 [N3+] - 9 [N3]
	63A GLU	2.26	11 [N3] - 627 [O.co2]
	87A GLN	3.21	876 [Nam] - 11 [N3]

Distance H-A: distance hydrogen/acceptor

The cofactor and ligand binding sites are composed principally of positively charged residues, indicating that it may preferably bind to negatively charged functional groups (Additional file 7). Structural superposition of cofactor bounded homology model of UGT2B10 with the crystal structure of UGT76G from *Stevia rebaudiana* (PDB ID: 6INF) revealed that the cofactor position is similar in both the cases, which indicate that the reliability of homology modeling as well as docking studies with the cofactor (Additional file 8).

### Molecular dynamics simulation

**Fig. 7:**
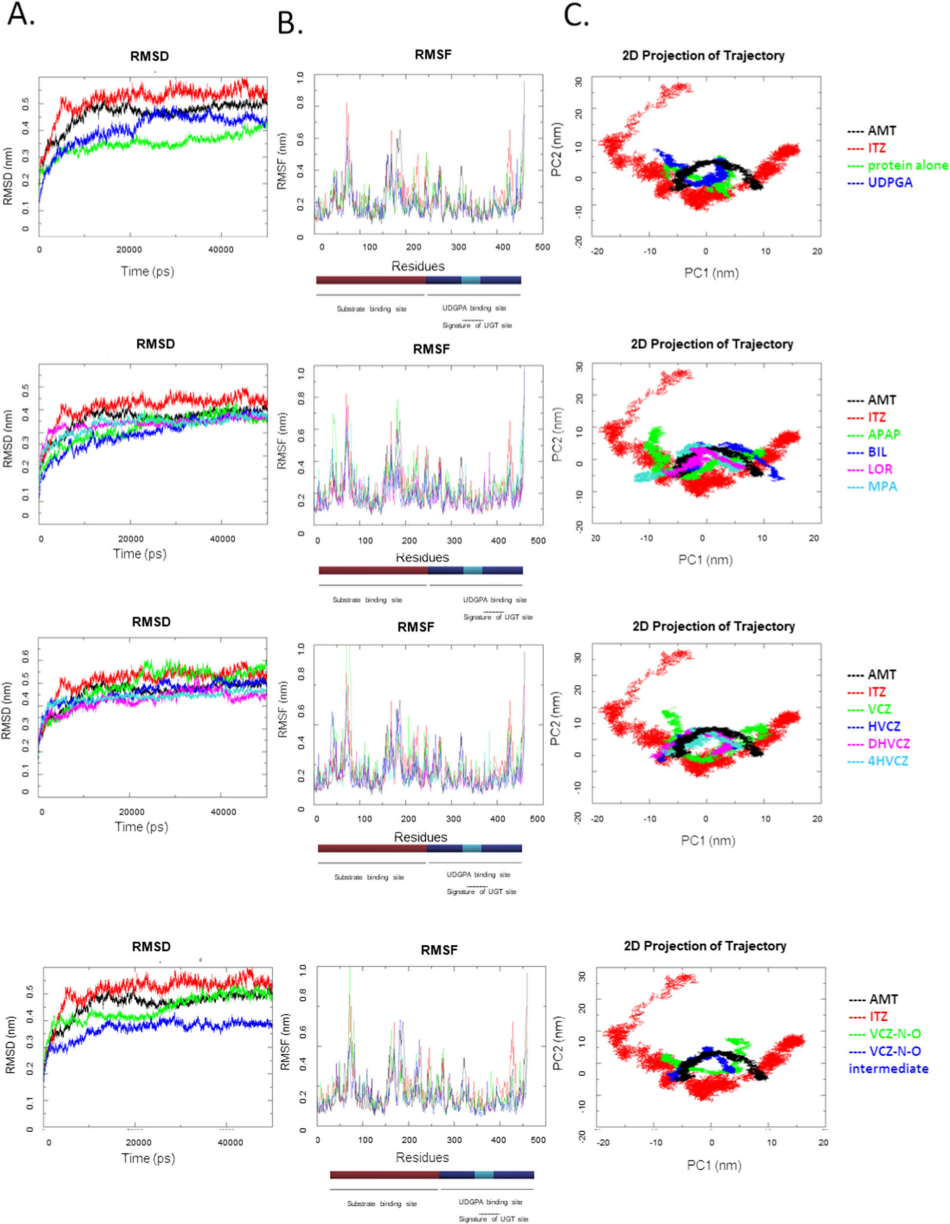
Molecular dynamics simulation results obtained from different UGT2B10 complexes. **A**) Root-mean square deviation **B**) Root mean square fluctuation **C**) Principal component analysis. The color code of ligands and ligands names are indicated on the plots

The results of the MD simulations are presented in the Table 5. Intramolecular hydrogen bonds were in the same range for all the tested complexes. The structure of UGT2B10-UDPGlcA has the higher average number of intermolecular hydrogen bonds after MD simulations (7.54 ± 2.01), indicating a stronger interaction between the protein and the co-factor, compared to the other tested ligands. The ligand with the highest average number of hydrogen bonds with the protein, is MPA. No hydrogen bond interactions were found in the complexes formed with BIL and 4HVCZ. The complex formed by VCZ-N-O intermediate UK-215,364 and UGT2B10 is predicted to have a good stability with an average root mean squared deviation (RMSD) value of 0.38 nm ± 0.03 and Radius of Gyration (RoG) of 2.3 nm ± 1.19*10^− 2^. For each complex, the part comprising the UDPGlcA binding site was more stable than the substrate binding site when looking at the RMSF values (Figure 7). Average root mean square fluctuation (RMSF) were consistent between the different complexes (Table 5). Other complexes formed with positive control AMT, LOR, MPA, HVCZ, DHVCZ, 4HVCZ, VCZ-N-O intermediate and APAP showed stability in a similar range (≤10% difference) (Figure 7, Table 5). The structure with the negative control ITZ is predicted to be the least stable regarding the results for RMSD and RoG, whereas the positive control AMT has a similar stability to the putative UGT2B10 substrates. Average SASA (solvent accessible surface areas) values were in the same range for all the complexes. ITZ showed the highest SASA value, indicating that a larger part of the protein is exposed to the solvent (244.84 nm^2^ ± 6.86) compared to the other tested molecules (Table 5). The trace of covariance matrix value was lower for VCZ-N-O intermediate, 4 and HVCZ compared to other complexes, indicating a higher stability for these complexes. In summary, the affinities of the selected ligands based on the structural stability (on the basis of essential motion) of the complexes for UGT2B10 is in the order of VCZ-N-O intermediate>4HVCZ > HVCZ>DHVCZ>LOR > AMT > VCZ-N-O > MPA > ITZ > BIL > APAP>VCZ. Regarding the binding free energy results, the Molecular Mechanics Poisson-Boltzmann Surface Area (MM-PBSA) analysis enabled to obtain negative binding free energy results with all the tested ligands, indicating that these ligands are likely to bind to UGT2B10. The predicted order of affinity was APAP > VCZ-N-O intermediate > LOR > AMT > MPA > ITZ > BIL > 4HVCZ > DHVCZ > HVCZ > VCZ > VCZ-N-O.

**Table 5:** Average values of hydrogen bonds, RMSD, RMSF, RoG, SASA, trace of the covariance matrix values and MM/PBSA binding free energy values of the different UGT2B10 with putative substrates

Complex	Average number of intra-molecular hydrogen bonds ± SD	Average number of inter-molecular hydrogen bonds ± SD	Average RMSD [nm ± SD]	Average RoG [nm]	Average RMSF [nm ± SD]	Average SASA [nm^**2**^ ± SD]	Trace of the covariance matrix [nm^**2**^]	MMPBSAbinding free energy [kcal/mol]
UGT2B10 apo form	302.73 ± 9.60	N/A	0.39 ± 0.05	2.26 ± 1.38*10^− 2^	0.20 ± 0.08	216.39 ± 4.74	42.08	NA
UGT2B10-UDPGlcA	305.29 ± 11.70	7.54 ± 2.01	0.43 ± 0.05	2.28 ± 9.57*10^− 4^	0.19 ± 0.09	221.01 ± 5.74	38.82	NC
UGT2B10-UDPGlcA-AMT	290.12 ± 10.25	0.21 ± 0.41	0.49 ± 0.05	2.29 ± 1.16*10^− 2^	0.21 ± 0.12	227.45 ± 4.47	55.6	− 160.85 ± 10.99
UGT2B10-UDPGlcA-APAP	304.67 ± 8.96	1.16 ± 0.68	0.47 ± 0.08	2.24 ± 2.84*10^− 2^	0.25 ± 0.11	224.61 ± 4.02	76.97	− 174.24 ± 13.38
UGT2B10-UDPGlcA-BIL	292.97 ± 11.69	0.00 ± 0.00	0.39 ± 0.07	2.31 ± 1.88*10^− 2^	0.21 ± 0.13	234.64 ± 3.93	65.10	−104.00 ± 11.06
UGT2B10-UDPGlcA-ITZ	310.11 ± 10.43	0.24 ± 0.44	0.51 ± 0.05	2.33 ± 1.29*10^− 2^	0.23 ± 0.14	244.84 ± 6.86	60.70	− 127.79 ± 15.25
UGT2B10-UDPGlcA-LOR	300.25 ± 8.69	0.65 ± 0.79	0.42 ± 0.06	2.28 ± 1.14*10^− 2^	0.20 ± 0.10	230.58 ± 3.67	48.81	− 162.07 ± 19.30
UGT2B10- UDPGlcA-MPA	303.7 ± 9.09	2.48 ± 1.33	0.47 ± 0.05	2.33 ± 1.21*10^− 2^	0.22 ± 0.16	230.48 ± 6.97	60.23	− 158.46 ± 11.95
UGT2B10-UDPGlcA-VCZ	301.51 ± 9.69	0.76 ± 0.96	0.49 ± 0.07	2.31 ± 2.16*10^− 2^	0.24 ± 0.16	234.23 ± 5.28	87.61	−59.56 ± 17.13
UGT2B10-UDPGlcA-HVCZ	311.18 ± 9.89	0.87 ± 0.66	0.46 ± 0.05	2.27 ± 1.13*10^− 2^	0.19 ± 0.09	224.92 ± 5.18	40.50	−85.43 ± 14.23
UGT2B10-UDPGlcA-DHVCZ	308.33 ± 9.97	1.21 ± 0.83	0.43 ± 0.04	2.29 ± 1.13*10^− 2^	0.20 ± 0.10	227.99 ± 4.71	43.27	−86.34 ± 25.35
UGT2B10-UDPGlcA-4HVCZ	309.36 ± 11.59	0.00 ± 0.00	0.44 ± 0.03	2.26 ± 8.81*10^− 3^	0.18 ± 0.09	223.49 ± 6.05	38.55	−95.43 ± 18.47
UGT2B10-UDPGlcA-VCZ-N-O	303.01 ± 10.28	1.38 ± 0.78	0.43 ± 0.05	2.30 ± 1.28*10^− 2^	0.22 ± 0.11	233.91 ± 5.20	58.16	− 12.41 ± 19.02
UGT2B10-UDPGlcA-VCZ-N-O-intermediate UK-215,364 [35]	305.54 ± 12.28	0.72 ± 0.52	0.38 ± 0.03	2.3 ± 1.19*10^− 2^	0.18 ± 0.1	227.28 ± 4.55	37.75	− 164.44 ± 13.38

Minimum distance analyses revealed that all the ligands are in close proximity (mean ± SD; 0.72 ± 0.33) to UDP except for BIL and 4HVCZ. It is important to note that VCZ and HVCZ exhibited proximity similar to that of AMT (mean ± SD; 0.298 ± 0.024 nm; Additional File 9).

## Discussion

In this report, we describe the first homology model for human UGT2B10. In addition, we successfully used this model for in silico protein-ligand interactions to predict putative ligands, providing evidence for the potential interaction of UGT2B10 with drugs and their metabolites used in pediatric hematopoietic stem cell transplantation (HSCT) setting with extensive molecular docking and MD simulation methods.

### Homology modelling of human UGT2B10 and docking predictions

As membrane proteins, human UGTs are difficult to crystallize and the few available structures consist only of the co-factor UDPGlcA binding site (*C*-terminal part of UGT2B7, residues 285–451 and UGT2B15, residues 284–451). We provided a method aimed to reduce time and resources allocated to define the protein structure of human UGT isoforms, by using a robust multiple template homology modelling approach (MODELLER, version 9.24). In silico methods were already used to build the 3D structures of some other human UGTs, but not UGT2B10 [50]. We provided the first homology model for the entire UGT2B10 protein, based on human, plant, and bacterial UGTs (model accessible on ModelArchive.org, DOI: https://modelarchive.org/doi/10.5452/ma-gx2va). A good affinity for the cofactor UDPGlcA was observed with a highly negative *ΔG* value, confirming the accuracy of the model. Besides, the positive control amitriptyline showed a high affinity towards UGT2B10 whereas the negative control itraconazole had a poor affinity, confirming the satisfactory prediction and reliability of the UGT2B10 structure.

### Molecular docking and MD simulations – putative UGT2B10 ligands

Semi flexible (protein is considered as rigid and ligand is considered as flexible) docking calculations are not sufficient for a reliable prediction of the binding affinities of putative ligands with proteins [51]. MD simulations, providing a dynamic environment and taking into consideration the change of conformation of the ligand and the protein, its evolution through time, and parameters such as temperature and pressure, is an essential complement to protein-ligand docking calculations [52]. These simulations provide more realistic energy predictions and parameters that are more comparable to in vivo conditions [53].

Based on the results obtained from MD simulations and MM-PBSA based binding free energy calculations, Acetaminophen (APAP), Lorazepam (LOR), Mycophenolic acid (MPA) and voriconazole-n-oxide (VCZ-N-O) intermediate are predicted to be putative ligands for human UGT2B10. Whereas other molecules such as HVCZ, DHVCZ, 4HVCZ and VCZ also reasonably fulfilled the criteria (stability and binding affinity) for being the ligands for UGT2B10. Based on the minimum distance analysis results we hypothesize that VCZ and HVCZ, could be the possible substrates of UGT2B10 since the computed distance values for these two ligands and UDP are comparable with the positive control AMT unlike other ligands that may serve as inhibitors. It is likely that the substrates for other UGT isoforms exhibiting sequence homology (e.g. UGT2B7) with that of UGT2B10 might also have higher affinities for the UGT2B10 substrate binding domain. Such ligands may serve as competitive inhibitors.

APAP is an analgesic antipyretic drug, known to cause acute hepatoxicity after intake of high doses, and in some cases even at therapeutic doses [54]. The majority of APAP (~ 95%) is degraded into non-toxic metabolites through sulfation or glucuronidation by UGT1A1, 1A6, 1A9, and 2B15 [42]. The rest of the parent compound (~ 5%) is metabolized by cytochrome P450, producing the hepatotoxic molecule *N*-acetyl-p-benzoquinone imine (NAPQI). Glutathione conjugation by glutathione-s-transferases further metabolize NAPQI to form non-toxic metabolites. Acute liver toxicity of APAP can arise from an overdose, saturating the GST conjugation pathway [55] but may also occur at therapeutic doses in case of hepatic function impairment [55]. For pediatric patients, the ontogeny of metabolic enzymes such as cytochrome or UGTs, is particularly important [56]. For example, younger patients are less prone to develop hepatotoxicity than adolescents, when exposed to a chronic dose of acetaminophen [56]. The UGT2B10 activity reaches the adult activity within a month after birth indicating its potential role in glucuronidation in infants and young children [57]. Meaning that the genetic variation is as important in children as it is in adults unlike other UGTs, e.g. UGT1A6 whose maximum activity is seen only in adolescence [57]. Variants located in 3′-UTR of the *UGT1A1* genes were demonstrated to influence APAP glucuronidation [58]. But no evidence was available about the contribution of UGT2B10 to the metabolism of APAP. However, UGTs form heterodimers with other close isoforms, substrate overlapping, or competitive inhibition cannot be ruled out among the members of this family. Significant correlation in the glucuronidation of the substrates of UGT2B10, UGT2B7 and UGT2B15 was observed, suggesting the substrate overlapping between these isoforms and possibly heterodimerization of the isoform’s units [17]. Given the concordance in the expression levels of UGT2B10, UGT2B7 and UGT2B15 also indicates that a genetic marker from this locus can also serve as a surrogate of the function of any of these isoforms [59]. The different levels of expression of UGT2B10, potentially influenced by the SNP rs17146905 may change the level of metabolism of APAP. Alternatively, the non-synonymous variant rs61750900 i.e. in strong LD with that of rs17146905 in Europeans and Americans could impact the function of UGT2B10 in carriers of rs17146905 leading to a potential increase of exposure to the hepatotoxic metabolites of APAP making more of APAP available for conversion into hepatotoxic metabolites by cytochrome P450 enzymes.

LOR is a benzodiazepine used as busulfan-related seizure prophylaxis in pediatric patient receiving a busulfan based conditioning regimens and is usually given from the day before the first busulfan infusion up to 24 h after the last dose [60]. This agent is cleared principally through glucuronidation. *R* and *S* enantiomers are degraded by different members of the UGTs enzymatic family. *S*-lorazepam is degraded by UGT2B4, 2B7, and 2B15, whereas *R*-lorazepam is metabolized by UGT2B4, 2B7, 2B15, 1A7, and 1A10 [37]. LOR contains an amine group, the chemical group catalyzed by UGT2B10 during *N*-glucuronidation. The drug class of benzodiazepine is rarely the cause of hepatotoxicity [61, 62]. In the case of lorazepam, the liver toxicity is also rare and there is only scarce observation of alanine aminotransferase (ALT) increase [63]. In addition, the usage of this drug is currently not reported as a risk factors for SOS, in pediatric HSCT patients [4]. It is worthy of note that Lorazepam is the only drug among the detected potential ligands, that is routinely co-administered with Busulfan. The other potential ligands that we describe, are not commonly administered at the same time as Busulfan.

MPA is the active metabolite of mycophenolate mofetil used as GvHD prophylaxis. Elevation of hepatic enzyme were reported in few patients receiving mycophenolate mofetil and liver toxicity is rarely reported [64]. Toxicity of mycophenolate mofetil in pediatric patients undergoing HSCT was reported once, and was probably due to multi-organ failure [65]. This active metabolite goes through metabolism principally by UGT1A8 and UGT1A9 to form an *O*-glucuronide conjugate [41]. UGT2B10 catalyzes mainly *N*-glucuronidation of tertiary amine compounds, while mycophenolic acid does not contain an amine group. It is also known that UGT2B7 partly contributes to the glucuronidation of MPA [17]. Thus, this molecule is not predicted to be a substrate for UGT2B10 but rather may act a competitive inhibitor. Consequently, it may modulate the enzymatic activity and the metabolism of other drugs.

As described above, LOR and MPA rarely cause severe hepatotoxicity, and are not predicted to be a risk factor for SOS. However, their combination with other known or potentially hepatotoxic chemotherapeutic and prophylactic agents in the HSCT setting, in addition to other preexisting risk factors—e.g. patients’ age, genetics, underlying disease, etc. [4, 66]—could potentiate the risk of occurrence of SOS in pediatric HSCT patients. It is also important to note that LOR and MPA could induce an elevation of the levels of hepatic enzymes in a small proportion of patients, and that increased transaminase levels are considered to be risk factors for SOS.

Voriconazole, the parent compound of VCZ-N-O intermediate and other VCZ metabolites investigated, is a known hepatotoxic molecule [67]. However, the correlation with the use of this antifungal and the occurrence of SOS in pediatric HSCT patients is not clearly established [4]. Furthermore, the direct hepatotoxicity of the metabolite VCZ-N-O intermediate and other VCZ metabolites and VCZ itself is yet to be studied. UGT1A4 was demonstrated to be involved in the degradation of voriconazole and some of its metabolites [35]. Interestingly, the UGT1A4 and UGT2B10 are the main catalyzer of *N*-glucuronidation and have overlapping substrates [20]. No experimental evidence was available on the potential role of UGT2B10 in the metabolism of these molecules, and an investigation in this regard may be interesting. VCZ-N-O intermediate, VCZ-N-O and other hydroxy metabolites undergo glucuronidation but the UGT isoform for this reaction are still unknown [34, 68]. UGT2B10 catalyzes principally *N*-glucuronidation and thus is not predicted to have VCZ metabolites as a substrate. Nevertheless, the high affinity between the putative ligand and UGT2B10 may indicate that VCZ-N-O intermediate can be a competitive inhibitor of UGT2B10, blocking the active site of the protein and reducing the catalytic activity. However, we may not rule out the involvement of UGTB10 in the O-glucuronidation reaction given the conserved residue D150, and owing functional evidence that UGT isoforms lacking H34 also facilitates O-glucuronidation e.g.UGT1A4 [69]. However, it is also worth noting that there is an experimental evidence indicating the importance of H34 in catalyzing O-glucuronidation reaction, that concluded the need for further investigations to elucidate its function [70]. It is also likely that binding affinities of the putative ligands may be biased by the template substrate specificities used for homology modelling as it was seen in case of UGT1A6 [71]. Since UGTs also participates in glucosidation, residues offering specificity for glucuronidation or glucosidation in N-terminal domain may also be biased by the template used for homology modelling(e.g. glucuronidation specificity offered by Arg259or glucosidation by UGT2B7) [72]. However homology modelling successfully implemented to test substrates and inhibitors of other UGT enzymes in the past with a satisfactory level of sensitivity and specificity [71].

APAP, LOR, MPA and VCN-N-O intermediate may thus either act as substrates or inhibitors, and could modulate the activity of UGT2B10, causing potential drug-drug interactions. This may be especially the case in the setting of conditioning regimens before HSCT, requiring a wide combination of drugs. Further in vitro enzymatic assays are required to confirm the role of these putative UGT2B10 ligands.

Another compound that was considered during the selection process of drugs and metabolites used during busulfan-based conditioning regimens before HSCT is the antifungal fluconazole (Figure 4). Fluconazole is principally eliminated through the renal route as a parent compound (> 80%) [73]. This compound was also demonstrated as an inhibitor of UGT2B10 in vitro, with an half inhibitory concentration (IC_50_) of 1.13 mM [25]. Meanwhile, there is currently no in vivo evidence of drug-drug interactions involving fluconazole through UGT2B10 metabolizing pathway. Interestingly, this antifungal can induce hepatotoxicity in pediatric patients [74]. This compound may also induce a different level of exposure to potential hepatotoxic molecules according to *UGT2B10* gene expression and function.

### MD simulation –with the endogenous compound bilirubin

Elevated bilirubin level is one of the Modified Seattle criteria [75], used to diagnose SOS in most of the reports including our EWAS report [10] in which the association between *UGT2B10* SNP and SOS was detected [10]. Interestingly, adults with Gilbert’s Syndrome were reported to have a higher occurrence of SOS following an HSCT [76]. The most common genotype of Gilbert’s syndrome is the homozygous, A (TA)7TAA *28 allele in the promoter of the *UGT1A1* gene, reducing the metabolism of bilirubin and causing an accumulation of unconjugated bilirubin in the blood [77]. UGT1A1 and UGT2B10 are not predicted to share the substrates as they share low sequence similarities and catalyzes mainly *O*- and *N*-glucuronidation, respectively. We nevertheless analyzed bilirubin as a substrate of UGT2B10. With AutoDock Vina, we found a *Kd* of 1.15*10^11^ μM for bilirubin, which shows that it is not predicted to be UGT2B10 substrate for. We further verified the results with MD simulation. Results indicated that the complex formed with bilirubin is stable, comparable to the stability of the complex formed with UGT2B10 and UDPGlcA alone (Additional file 6**,** Additional file 8), meaning that the outcome was contradictory with that of the molecular docking since it is based on semi flexible approach). Further an in vitro elucidation on the role of UGT2B10 on bilirubin metabolism is thus warranted, amidst the recent evidence on the role of *UGT2B10* variant on altered bilirubin levels in a recent longitudinal study [78].

### Methodology – advantages and limitations

Our method provides a rational way to select interesting putative substrates to be tested further in in vitro enzyme assays. This method saves time and is cost-effective. Computational tools used are freely available and user friendly, such as AutoDock Vina (version 1.1.2) [47] and MODELLER (version 9.24) [79]. MD simulations in contrast (GROMACS) (version 5.1.4)) [80]) require more resources and expertise, and must be performed in collaboration with a high-performance computing facility to reduce the simulation time. Nevertheless, these analyses can be achieved in a reasonable time, and can refine follow-up in vitro assays reducing the costs associated with testing of multiple molecules.

One of the limitations of our study is on the selection criteria of putative UGT2B10 ligands to be tested with MD simulations. As MD is a time-consuming process, we used a strict threshold for the selection of complexes to test protein-ligand docking with AutoDock Vina. Thus, we choose to base the filtering on the *ΔG* values obtained with positive control AMT. We used a strict threshold based on the results of the known ligand amitriptyline. Thus, we may have excluded potential false negative results, showing less affinity with the UGT2B10 but still interacting as ligands.

On the other hand, this method only predicts molecules interacting with the protein. The stability of the complex and energies can be predicted, but we cannot determine if the molecule undergoes glucuronide conjugations. Besides of some assumptions made based on the chemical structure, there is no possibility to determine if the molecule acts as a substrate, an inhibitor, or an inducer of UGT2B10. To have more insight into the precise role of the molecule identified, further in vitro experiments are needed to characterize the products or measure the effect of the ligand on the UGT2B10 activity. In our analysis, we chose to perform site-specific docking to focus on the detection of potential substrates while also screening for potential competitive inhibitors. The methodology used provides site-specific interaction with the putative ligands, not enabling to detect another mode of inhibition such as non-competitive inhibition, where the binding of the inhibitor does not occur in the active site of the protein. To detect potential non-competitive inhibitors, blind or non-specific docking studies can be performed to define where the interaction is most likely to occur on the protein.

Further in vitro assays must be performed in order to confirm in silico predictions and test the validity of their results. Developed enzymatic assays using recombinant UGT2B10 coupled with liquid chromatography-tandem mass spectrometry analytical methods [20, 57, 81, 82] could be implemented to screen our test compounds as substrates or inhibitors of UGT2B10.

## Conclusion

Using a step-wise methodology, we provide a systematic approach for the exploration of the potential substrates of a drug-metabolizing enzyme in silico. This enables prioritization for follow-up in vitro experiments allowing optimal utilization of time and resources. The methodology is flexible, and any other interesting proteins or ligands can be added to the selection pipeline.

We provided the first complete human UGT2B10 modeled structure. With a selection pipeline of drugs used during busulfan-based conditioning regimens, we detected potential interesting UGT2B10 ligands; acetaminophen, lorazepam, mycophenolic acid, and voriconazole-n-oxide intermediate. These compounds could be tested with enzyme kinetics followed by LC-MS/MS experiments to determine if the ligands are putative inhibitors or substrates.

## Methods

### Generation of the UGT2B10 model

Template-based or homology or comparative protein modelling of UGT2B10 was performed with MODELLER (version 9.24) [79]. The amino acid sequence was retrieved from UniProt protein sequence database with the accession number of P36537 [83]. The signal peptide was removed, as it only serves to direct the protein to the endoplasmic reticulum and is not present in the mature protein. The HHpred tool [26, 27] was used to search homologous proteins from the protein databank (PDB) repository. Briefly, a multi-sequence alignment (MSA) is built with a homologous sequence from the Non-redundant protein sequence (NRDB) database. A hidden-Markov model (HMM) profile is created based on this MSA, and the RCSB-PDB database is scanned for similar HMM profiles. Potential templates or structural neighbors retrieved by HHpred were filtered to include only protein structures with more than 20% similarity with the query sequence, obtained by X-ray crystallography, of a resolution of less than 2 Å and with UDP-binding domains [84] verified manually with InterPro [85]. Multiple templates were selected to provide a good quality model [86]. The four selected templates, UTG51 from *S. cerevisiae* (PDB ID: 5GL5), UGT76G from *S. rebaudiana* (PDB ID: 6INF), UGT2B7 from *H. sapiens* (PDB ID: 2O6L), and oleodamycin glycosyltransferase from *S. antibioticus* (PDB ID: 2IYA) (Table 1) were used to build the MSA. The alignment was submitted to the MODELLER tool [87] provided in the Chimera software (version 1.15) [87] to construct the homology model. The results with the more negative normalized Discrete Optimized Protein Energy (zDOPE) score were selected as the best model. Structure of the protein and protein-ligand complexes were analyzed with PDBsum [49]. Apart from this, we also retrieved the structure of UGTB10 from AlphaFold [88], an in silico protein structure database, which was developed very recently.

### Quality of the model and energy minimization

Energy minimization of our UGT2B10 model was performed first with Yasara minimization server [29] and when the quality was no more improved with GalaxyRefine, until the maximum quality was reached [28]. The final model was obtained after three steps of each respective tool **(**Additional file 3**)** The quality of the model was assessed with Verify3D [31, 32], ERRAT [30], and Ramachandran plot, by using the Structure Analysis and Verification (SAVES) servers [89]. Z-score was obtained with the ProSA-web server [33] (Additional file 2). Further, we compared the structure validation results of our model with model by AlphaFold. The structure superposition between our homology model and AlphaFold model was also attempted using PYMOL [88]. Finally, PDBSum Generate server was used to compute the secondary structure composition of both AlphaFold and our homology models.

### Binding site prediction

Binding site prediction was performed with MetaPocket 2.0, a consensus-based method [90]. Results from LIGSITES, PASS, Q-SiteFinder, SURFNET, Fpocket, GHECOM, ConCavity and POCASA are compiled with this tool [90]. Prediction returned by many of the tools was determined as the best results. The prediction was further refined and validated after the superposition of the templates with our homology model using PyMol [91]. In addition, Comparative protein sequence alignment using Clustal Omega (https://www.ebi.ac.uk/Tools/msa/clustalo/) was also performed for all the members of UGT to identify the putative catalytic residues of UGT2B10.

### Molecular docking with cofactor UDPGlcA

Binding with the cofactor UDPGlcA was performed with the AutoDock Vina docking algorithm [92]. Simplified Molecular Input Line Entry System (SMILES) structure of UDPGlcA was retrieved from PubChem (CID: 17473) and then saved in a PDB format with Corina 3D structure conversion web server [93]. The UGT2B10 model and the ligand were prepared with Molecular Graphics Laboratory (MGL) Tools (version 1.5.7) [94]. In this step, the polar hydrogens were added, and non-polar hydrogens were merged in both protein and ligand structures. Moreover, the kollmann charges was added to the protein whereas Gasteiger charges was added to ligand structures. Finally, the protein and ligand structures were recorded into in PDBQT format (XYZ Coordinates + Partial charges + atom type). The receptor grid box had a size of was restricted to the previously defined (size (x,y,z) = (15, 17, 22.5) and center coordinates of (x,y,z) = (− 70, 8, 3)). A maximum of nine binding modes for each ligand were returned by the tool. Results with the more negative *ΔG* (in kcal/mol) were selected as the best docking conformation.

### Consensus based approach for selection of putative UGT2B10 ligands

A list of drugs used during and after busulfan-based conditioning regimens for HSCT in pediatric was created, based on the discovery cohort of our EWAS study [10]. This list included chemotherapeutics, antifungals, SOS and GvHD prophylaxis, and antipyretics. Each molecule passed through a pipeline to choose the most interesting compounds to be tested for molecular docking calculations with UGT2B10 (Figure 4). The approach consisted of receiving inputs on inclusion and exclusion criteria independently from four individuals with background in pediatric oncology, pharmacology and biology. These criteria included clinical relevance in pediatric HSCT setting (such as the frequency of usage), in addition to verification of previous in vitro and in vivo evidence regarding glucuronidation. 14 drugs were relevant drugs used in pediatric HSCT setting were found. From the list three were excluded because there was already evidence that the molecules do not undergo glucuronidation in vivo. Relevant metabolites for these drugs were added to the selection pipelines, for a total of 11 drugs and 7 degradation products. From these 18 molecules, three were further excluded because they were not directly metabolized by UGTs, and were already demonstrated to not undergo glucuronidation in vitro (Figure 4). Based on the consensus from the above-mentioned criteria, selected ligands were chosen further for in silico protein-ligand interactions using molecular docking and MD simulations.

Molecular docking was performed with the same method as for the cofactor UDPGlcA using AutoDock Vina docking algorithm [92]. Amitriptyline and itraconazole were used as positive and negative controls, respectively [25]. The affinity between the ligand and UGT2B10 was quantified after calculation of the dissociation constant (*Kd*) (Equation 1) [95].

1 $$ Kd\ (M)={e}^{\frac{\varDelta G}{R\ast T}} $$

Where *ΔG* is the estimated free energy of binding in kcal/mol, R the gas constant (1.957 * 10^− 3^ kcal*K^− 1^*mol^− 1^), and T the temperature in Kelvin (298 K for an experiment conducted at 25°). A low Kd indicates a high affinity between the ligand and the enzyme. Further, molecular docking studies were also performed for AlphaFold UGTB10 model with the selected ligands and compared the results obtained from our homology model of UGTB10.

### Molecular dynamics simulations with GROMACS

To understand the structural stability and interaction between enzyme and putative ligands, the molecular dynamics (MD) simulation was performed with the time period of 50 ns at the University of Geneva on the Baobab cluster with GROMACS (version 5.1.4) [80] on the structures obtained after molecular docking calculation. Eight compounds showing *ΔG* value of ≤ − 1.0 kcal/mol predicted by AutoDock Vina (based on the results obtained for the positive control amitriptyline (Table 3)), the negative and positive controls (ITZ and AMT), the protein with only the cofactor UDPGlcA and the apo form were selected to perform these simulations. Every structure composed of a putative ligand, UGT2B10 and UDPGlcA was compared with the omplex comprising only UGT2B10 and UDPGlcA. The process of the MD simulation was based on the work of Lemkul et al. [96] Briefly, enzyme structure was prepared with The GROMOS force field set 53a6 [97]. The topology (ITP) file and the GROMACS (GRO) file for the ligand structures were prepared with the external PRODRG server [98]. The system temperature was set to 310 K (36.9 °C), and the reference pressure to 1.0 bar. The LINCS constraint solver algorithm was used on all bonds with “lincs_iter” and “lincs_order” parameters of 1 and 4 respectively. The short range electrostatic and van der Waals cutoffs were both set to 1.0 nm. The MD simulations were run for a period of 50 ns with a step size of 2 fs. The analysis of the results was first performed with GROMACS functions to provide the principal component analysis (PCA) of the projection of trajectories of the backbone of the protein, root means square fluctuation (RMSF; Gromacs command: *gmx rmsf*) and root mean square deviation (RMSD; Gromacs command: *gmx rms*) graphs. The average number of inter-and intra- molecular H-bonds (Gromacs command: *gmx hbonds*), RMSD, RMSF, Radius of Gyration (RoG; Gromacs command: *gmx gyrate*), and Solvent Accessible Surface Area (SASA) were calculated with the average command from GROMACS. The trace of covariance matrix was determined with the *gmx covar* and *gmx anaeag* commands. To evaluate the proximity of the ligands including positive (AMT) and negative controls (ITZ) towards cofactor, we estimated minimum distance analysis using gmx mindist module of Gromacs, To have quantitative results, further MM-PBSA based calculation of the binding free energies was performed with *g_mmpbsa* version 1.6 [99]. MM-PBSA calculations were performed on the most stable portion of the simulation for each complex, corresponding to the last 20 ns i.e. from 30 to 50 ns when the complex is more stable (2000 frames with increments every 10 ps). Results and graphs were presented with Chimera version 1.14 and QTGrace version 0.2.6. A complete workflow of the molecular docking and MD simulations is presented in Additional file 10.

A detailed step-wise protocol followed for molecular dynamics simulations is available publicly from the Yareta repository (DOI: 10.26037/yareta:o7au7wijvrglnb6nu2k4azkmmy). A list of the tools and software used can be found in the supplementary materials (Additional file 11**).**

## Supplementary Information

**Additional file 1.** List of in vitro and in vivo UGT2B10 drug ligands. IC50: half inhibitory concentration; Ki: inhibitory constant; Km: Michaelis constant; N/A: not applicable.

**Additional file 2.** Quality of the final UGT2B10 and AlphaFold model, based on ERRAT, Verify3D, ProSA and Ramachandran plots. Comparison with AlphaFold structure is also given in the table.

**Additional file 3.** Secondary structure of UGT2B10 obtained with PDBsum.

**Additional file 4.** Predicted A) cofactor binding site, B) substrate-binding site of human UGT2B10 model (The green box represents the grid box for the ligand docking simulation with AutoDock Vina), C) Structural superposition analysis of our model with the AlphaFold Model (Display: PyMOL) Red: AlphaFold; Blue: Our Model.

**Additional file 5.** Structures of the selected molecules to perform the molecular docking.

**Additional file 6.** Comparative molecular docking results of refined model of UGTB10 and AlphaFold model with the selected ligands.

**Additional file 7.** Examples of electrostatic graphs of UGT2B10 with the tested ligands. A) UDPGlcA B) amitriptyline C) lorazepam. Red indicates negatively charged amino acid residues and blue positively charged amino acid residues. White indicates neutral residues.

**Additional file 8 **Identification of cofactor location of UGT2B10 Model (by superposition with UGT76G from *Stevia rebaudiana* (PDB ID: 6INF) (Color codes: Blue: UDP orientation in UGT2B10 and Magenta: UGP orientation in crystal structure).

**Additional file 9.** Domain distance analysis of UDP with various ligands (a): AMT: RED, ITZ: BLUE, BIL: YELLOW, VCZN: GREEN, VCZ: ORANGE. (b) AMT: RED, ITZ: BLUE, MPA: CYAN, APAP: MAGENTA, LORAZEPAM: DARK GREEN and (c): AMT: RED, ITZ: BLUE, DHVCZ: PURPLE, HVCZ: GOLD, 4HVCZ: OLIVE

**Additional file 10.** Workflow of molecular docking predictions and molecular dynamics simulations performed to find potential UGT2B10 ligands.

**Additional file 11.** List of tools, software and websites used for multiple template homology modeling of human UGT2B10, docking predictions and MD simulations.

## Data Availability

PDB file of human UGT2B10 is available from ModelArchive.org (https://modelarchive.org/doi/10.5452/ma-gx2va). The MD simulation data and the MD simulations procedure are publicly available on the Yareta repository from the University of Geneva. (DOI: 10.26037/yareta:o7au7wijvrglnb6nu2k4azkmmy).

## Abbreviations

4HVCZ: 4-hydroxyvoriconazole
AMT: Amitriptyline
APAP: Acetaminophen
DHVCZ: Dihydroxy voriconazole
EWAS: Exome-wide association study
HVCZ: Hydroxy voriconazole
GvHD: Graft versus host disease
HMM: Hidden Markov models
HSCT: Hematopoietic stem cell transplantation
IC_50_: Half inhibitory concentration
ITZ: Itraconazole
*Kd*: Dissociation constant
LOR: Lorazepam
MD: Molecular dynamics
miRNA: Micro RNA
MPA: Mycophenolic acid
NAPQI: N-acetyl-p-benzoquinone
NPT: Number of particles, pressure, and temperature
NRDB: Non-redundant protein sequence database
NVT: constant number of particles, volume, and temperature
RMSD: Root mean square deviation
RMSF: Root mean square fluctuation
mRNA: Messenger RNA
RoG: Radius of gyration
MSA: Multiple sequence alignment
PDB: Protein databank
PCA: principal component analysis
SASA: Solvent accessible surface area
SD: Standard deviation
SMILES: Simplified Molecular Input Line Entry System
SNP: Single nucleotide polymorphism
SOS: Sinusoidal occlusion syndrome
UDPGlcA: UDP-glucuronic acid
UGT: UDP-glucuronosyltransferase
UTR: Untranslated region
VCZ-N-O: Voriconazole N-oxide
VCZ-N-O-intermediate: Voriconazole N-oxide intermediate

## References

[1] Corbacioglu S, Carreras E, Ansari M, Balduzzi A, Cesaro S, Dalle J-H, Dignan F, Gibson B, Guengoer T, Gruhn B, Lankester A, Locatelli F, Pagliuca A, Peters C, Richardson PG, Schulz AS, Sedlacek P, Stein J, Sykora KW, Toporski J, Trigoso E, Vetteranta K, Wachowiak J, Wallhult E, Wynn R, Yaniv I, Yesilipek A, Mohty M, Bader P (2018). Diagnosis and severity criteria for sinusoidal obstruction syndrome/veno-occlusive disease in pediatric patients: a new classification from the European society for blood and marrow transplantation. Bone Marrow Transplant.

[2] Kammersgaard MB, Kielsen K, Heilmann C, Ifversen M, Müller K (2019). Assessment of the proposed EBMT pediatric criteria for diagnosis and severity grading of sinusoidal obstruction syndrome. Bone Marrow Transplant.

[3] Huezo-Diaz Curtis P, Uppugunduri CRS, Muthukumaran J, Rezgui MA, Peters C, Bader P (2018). Association of CTH variant with sinusoidal obstruction syndrome in children receiving intravenous busulfan and cyclophosphamide before hematopoietic stem cell transplantation. Pharmacogenomics J.

[4] Corbacioglu S, Jabbour EJ, Mohty M (2019). Risk factors for development of and progression of hepatic Veno-occlusive disease/sinusoidal obstruction syndrome. Biol Blood Marrow Transplant J Am Soc Blood Marrow Transplant.

[5] Ansari M, Curtis PH-D, Uppugunduri CRS, Rezgui MA, Nava T, Mlakar V, Lesne L, Théoret Y, Chalandon Y, Dupuis LL, Schechter T, Bartelink IH, Boelens JJ, Bredius R, Dalle JH, Azarnoush S, Sedlacek P, Lewis V, Champagne M, Peters C, Bittencourt H, Krajinovic M (2017). GSTA1 diplotypes affect busulfan clearance and toxicity in children undergoing allogeneic hematopoietic stem cell transplantation: a multicenter study. Oncotarget..

[6] Ansari M, Huezo-Diaz P, Rezgui MA, Marktel S, Duval M, Bittencourt H, Cappelli B, Krajinovic M (2016). Influence of glutathione S -transferase gene polymorphisms on busulfan pharmacokinetics and outcome of hematopoietic stem-cell transplantation in thalassemia pediatric patients. Bone Marrow Transplant.

[7] Huezo-Diaz P, Uppugunduri Satyanarayana CR, Tyagi AK, Krajinovic M, Ansari Djaberi MG (2014). Pharmacogenetic aspects of drug metabolizing enzymes in busulfan based conditioning prior to allogenic hematopoietic stem cell transplantation in children. Curr Drug Metab.

[8] Bonifazi F, Barbato F, Ravaioli F, Sessa M, Defrancesco I, Arpinati M, Cavo M, Colecchia A (2020). Diagnosis and treatment of VOD/SOS after allogeneic hematopoietic stem cell transplantation. Front Immunol.

[9] Cairo MS, Cooke KR, Lazarus HM, Chao N (2020). Modified diagnostic criteria, grading classification and newly elucidated pathophysiology of hepatic SOS/VOD after haematopoietic cell transplantation. Br J Haematol.

[10] Ansari M, Petrykey K, Rezgui MA, Del Vecchio V, Cortyl J, Ralph R-O (2020). Genetic susceptibility to hepatic sinusoidal obstruction syndrome in pediatric patients undergoing hematopoietic stem cell transplantation. Biol Blood Marrow Transplant J Am Soc Blood Marrow Transplant.

[11] Mayr C (2018). What are 3′ UTRs doing?. Cold Spring Harb Perspect Biol.

[12] Dluzen DF, Sutliff AK, Chen G, Watson CJW, Ishmael FT, Lazarus P (2016). Regulation of UGT2B expression and activity by miR-216b-5p in liver Cancer cell lines. J Pharmacol Exp Ther.

[13] Rowland A, Miners JO, Mackenzie PI (2013). The UDP-glucuronosyltransferases: their role in drug metabolism and detoxification. Int J Biochem Cell Biol.

[14] Fujiwara R, Yokoi T, Nakajima M. Structure and protein–protein interactions of human UDP-glucuronosyltransferases. Front Pharmacol. 2016;7. 10.3389/fphar.2016.00388. 10.3389/fphar.2016.00388 PMC5075577 27822186

[15] Meech R, Miners JO, Lewis BC, Mackenzie PI (2012). The glycosidation of xenobiotics and endogenous compounds: versatility and redundancy in the UDP glycosyltransferase superfamily. Pharmacol Ther.

[16] Allain EP, Rouleau M, Lévesque E, Guillemette C (2020). Emerging roles for UDP-glucuronosyltransferases in drug resistance and cancer progression. Br J Cancer.

[17] Badée J, Qiu N, Collier AC, Takahashi RH, Forrest WF, Parrott N, Schmidt S, Fowler S (2019). Characterization of the ontogeny of hepatic UDP-glucuronosyltransferase enzymes based on Glucuronidation activity measured in human liver Microsomes. J Clin Pharmacol.

[18] Couto N, Al-Majdoub ZM, Achour B, Wright PC, Rostami-Hodjegan A, Barber J (2019). Quantification of proteins involved in drug metabolism and disposition in the human liver using label-free global proteomics. Mol Pharm.

[19] Kato Y, Izukawa T, Oda S, Fukami T, Finel M, Yokoi T, Nakajima M (2013). Human UDP-glucuronosyltransferase (UGT) 2B10 in drug N-glucuronidation: substrate screening and comparison with UGT1A3 and UGT1A4. Drug Metab Dispos Biol Fate Chem..

[20] Lu D, Xie Q, Wu B (2017). N-glucuronidation catalyzed by UGT1A4 and UGT2B10 in human liver microsomes: assay optimization and substrate identification. J Pharm Biomed Anal.

[21] Chen G, Dellinger RW, Gallagher CJ, Sun D, Lazarus P (2008). Identification of a prevalent functional missense polymorphism in the UGT2B10 gene and its association with UGT2B10 inactivation against tobacco-specific nitrosamines. Pharmacogenet Genomics.

[22] Kaivosaari S, Toivonen P, Hesse LM, Koskinen M, Court MH, Finel M (2007). Nicotine glucuronidation and the human UDP-glucuronosyltransferase UGT2B10. Mol Pharmacol.

[23] Turgeon D, Chouinard S, Belanger P, Picard S, Labbe J-F, Borgeat P (2003). Glucuronidation of arachidonic and linoleic acid metabolites by human UDP-glucuronosyltransferases. J Lipid Res.

[24] Myers AL, Kawedia JD, Champlin RE, Kramer MA, Nieto Y, Ghose R, Andersson BS (2017). Clarifying Busulfan metabolism and drug interactions to support new therapeutic drug monitoring strategies: a comprehensive review. Expert Opin Drug Metab Toxicol.

[25] Pattanawongsa A, Nair PC, Rowland A, Miners JO (2016). Human UDP-glucuronosyltransferase (UGT) 2B10: validation of cotinine as a selective probe substrate, inhibition by UGT enzyme-selective inhibitors and antidepressant and antipsychotic drugs, and structural determinants of enzyme inhibition. Drug Metab Dispos Biol Fate Chem..

[26] Söding J, Biegert A, Lupas AN (2005). The HHpred interactive server for protein homology detection and structure prediction. Nucleic Acids Res.

[27] Zimmermann L, Stephens A, Nam S-Z, Rau D, Kübler J, Lozajic M, Gabler F, Söding J, Lupas AN, Alva V (2018). A completely Reimplemented MPI bioinformatics toolkit with a new HHpred server at its Core. J Mol Biol.

[28] Heo L, Park H, Seok C (2013). GalaxyRefine: protein structure refinement driven by side-chain repacking. Nucleic Acids Res.

[29] Krieger E, Joo K, Lee J, Lee J, Raman S, Thompson J, Tyka M, Baker D, Karplus K (2009). Improving physical realism, stereochemistry, and side-chain accuracy in homology modeling: four approaches that performed well in CASP8. Proteins..

[30] Colovos C, Yeates TO (1993). Verification of protein structures: patterns of nonbonded atomic interactions. Protein Sci Publ Protein Soc.

[31] Bowie JU, Lüthy R, Eisenberg D (1991). A method to identify protein sequences that fold into a known three-dimensional structure. Science..

[32] Lüthy R, Bowie JU, Eisenberg D (1992). Assessment of protein models with three-dimensional profiles. Nature..

[33] Wiederstein M, Sippl MJ (2007). ProSA-web: interactive web service for the recognition of errors in three-dimensional structures of proteins. Nucleic Acids Res.

[34] Roffey SJ, Cole S, Comby P, Gibson D, Jezequel SG, Nedderman ANR, Smith DA, Walker DK, Wood N (2003). The disposition of Voriconazole in mouse, rat, rabbit, Guinea pig, dog, and human. Drug Metab Dispos.

[35] Bourcier K, Hyland R, Kempshall S, Jones R, Maximilien J, Irvine N, Jones B (2010). Investigation into UDP-glucuronosyltransferase (UGT) enzyme kinetics of imidazole- and Triazole-containing antifungal drugs in human liver Microsomes and recombinant UGT enzymes. Drug Metab Dispos.

[36] Strassburg CP, Barut A, Obermayer-Straub P, Li Q, Nguyen N, Tukey RH, Manns MP (2001). Identification of cyclosporine a and tacrolimus glucuronidation in human liver and the gastrointestinal tract by a differentially expressed UDP-glucuronosyltransferase: UGT2B7. J Hepatol.

[37] Uchaipichat V, Suthisisang C, Miners JO (2013). The glucuronidation of R- and S-lorazepam: human liver microsomal kinetics, UDP-glucuronosyltransferase enzyme selectivity, and inhibition by drugs. Drug Metab Dispos Biol Fate Chem.

[38] Widemann BC, Sung E, Anderson L, Salzer WL, Balis FM, Monitjo KS, McCully C, Hawkins M, Adamson PC (2000). Pharmacokinetics and metabolism of the methotrexate metabolite 2, 4-diamino-N (10)-methylpteroic acid. J Pharmacol Exp Ther.

[39] Vree TB, Lagerwerf AJ, Verwey-van Wissen CP, Jongen PJ (1999). High-performance liquid chromatography analysis, preliminary pharmacokinetics, metabolism and renal excretion of methylprednisolone with its C6 and C20 hydroxy metabolites in multiple sclerosis patients receiving high-dose pulse therapy. J Chromatogr B Biomed Sci App.

[40] DiFrancesco R, Frerichs V, Donnelly J, Hagler C, Hochreiter J, Tornatore KM (2007). Simultaneous determination of cortisol, dexamethasone, methylprednisolone, prednisone, prednisolone, mycophenolic acid and mycophenolic acid glucuronide in human plasma utilizing liquid chromatography–tandem mass spectrometry. J Chromatogr B.

[41] Bernard O, Guillemette C (2004). The main role of UGT1A9 in the hepatic metabolism of mycophenolic acid and the effects of naturally occurring variants. Drug Metab Dispos Biol Fate Chem..

[42] Mutlib AE, Goosen TC, Bauman JN, Williams JA, Kulkarni S, Kostrubsky S (2006). Kinetics of acetaminophen glucuronidation by UDP-glucuronosyltransferases 1A1, 1A6, 1A9 and 2B15. Potential implications in acetaminophen-induced hepatotoxicity. Chem Res Toxicol.

[43] Ghosal A, Hapangama N, Yuan Y, Achanfuo-Yeboah J, Iannucci R, Chowdhury S, Alton K, Patrick JE, Zbaida S (2004). Identification of human UDP-glucuronosyltransferase enzyme(s) responsible for the glucuronidation of posaconazole (Noxafil). Drug Metab Dispos Biol Fate Chem..

[44] Zhou D, Kong L, Jiang Y, Wang C, Ni Y, Wang Y, Zhang H, Ruan J (2019). UGT-dependent regioselective glucuronidation of ursodeoxycholic acid and obeticholic acid and selective transport of the consequent acyl glucuronides by OATP1B1 and 1B3. Chem Biol Interact.

[45] Categorization Of The Likelihood Of Drug Induced Liver Injury (2012). In: LiverTox: clinical and research information on drug-induced liver injury.

[46] Senior AW, Evans R, Jumper J, Kirkpatrick J, Sifre L, Green T, Qin C, Žídek A, Nelson AWR, Bridgland A, Penedones H, Petersen S, Simonyan K, Crossan S, Kohli P, Jones DT, Silver D, Kavukcuoglu K, Hassabis D (2020). Improved protein structure prediction using potentials from deep learning. Nature..

[47] Trott O, Olson AJ (2010). AutoDock Vina: improving the speed and accuracy of docking with a new scoring function, efficient optimization and multithreading. J Comput Chem.

[48] SMILES explorer. http://www.cheminfo.org/Chemistry/Cheminformatics/Smiles/index.html. Accessed 7 Jan 2021.

[49] Laskowski RA, Jabłońska J, Pravda L, Vařeková RS, Thornton JM (2018). PDBsum: structural summaries of PDB entries. Protein Sci Publ Protein Soc.

[50] Nair PC, Meech R, Mackenzie PI, McKinnon RA, Miners JO (2015). Insights into the UDP-sugar selectivities of human UDP-glycosyltransferases (UGT): a molecular modeling perspective. Drug Metab Rev.

[51] Chen Y-C (2015). Beware of docking!. Trends Pharmacol Sci.

[52] Hospital A, Goñi JR, Orozco M, Gelpí JL (2015). Molecular dynamics simulations: advances and applications. Adv Appl Bioinforma Chem AABC.

[53] Sakano T (2016). Mahamood MdI, Yamashita T, Fujitani H. molecular dynamics analysis to evaluate docking pose prediction. Biophys Physicobiology.

[54] Larson AM (2007). Acetaminophen hepatotoxicity. Clin Liver Dis.

[55] McClain CJ, Price S, Barve S, Devalarja R, Shedlofsky S (1999). Acetaminophen hepatotoxicity: an update. Curr Gastroenterol Rep.

[56] Krasniak AE, Knipp GT, Svensson CK, Liu W. Pharmacogenomics of acetaminophen in pediatric populations: a moving target. Front Genet. 2014;5. 10.3389/fgene.2014.00314. 10.3389/fgene.2014.00314 PMC4196544 25352860

[57] Badée J, Qiu N, Parrott N, Collier AC, Schmidt S, Fowler S (2019). Optimization of experimental conditions of automated Glucuronidation assays in human liver Microsomes using a cocktail approach and ultra-high performance liquid chromatography-tandem mass spectrometry. Drug Metab Dispos Biol Fate Chem.

[58] Mehboob H, Tahir IM, Iqbal T, Saleem S, Perveen S, Farooqi A (2017). Effect of UDP-glucuronosyltransferase (UGT) 1A polymorphism (rs8330 and rs10929303) on Glucuronidation status of acetaminophen. Dose-Response Publ Int Hormesis Soc.

[59] Badée J, Fowler S, de Wildt SN, Collier AC, Schmidt S, Parrott N (2019). The ontogeny of UDP-glucuronosyltransferase enzymes, recommendations for future profiling studies and application through physiologically based pharmacokinetic modelling. Clin Pharmacokinet.

[60] Zao JH, Schechter T, Liu WJ, Gerges S, Gassas A, Egeler RM, Grunebaum E, Dupuis LL (2015). Performance of Busulfan dosing guidelines for pediatric hematopoietic stem cell transplant conditioning. Biol Blood Marrow Transplant.

[61] Björnsson E (2008). Hepatotoxicity associated with antiepileptic drugs. Acta Neurol Scand.

[62] Chalasani N, Fontana RJ, Bonkovsky HL, Watkins PB, Davern T, Serrano J (2008). Causes, clinical features, and outcomes from a prospective study of drug-induced liver injury in the United States. Gastroenterology.

[63] Arai T, Kogi K, Honda Y, Suzuki T, Kawai K, Okamoto M, Fujioka T, Murata N (2018). Lorazepam as a cause of drug-induced liver injury. Case Rep Gastroenterol.

[64] Hoofnagle JH, Serrano J, Knoben JE, Navarro VJ (2013). LiverTox: a website on drug-induced liver injury. Hepatology..

[65] Basara N, Fauser AA (2000). Safety profile of mycophenolate mofetil. Bone Marrow Transplant.

[66] Dalle J-H, Giralt SA (2016). Hepatic Veno-occlusive disease after hematopoietic stem cell transplantation: risk factors and stratification, prophylaxis, and treatment. Biol Blood Marrow Transplant J Am Soc Blood Marrow Transplant.

[67] Xing Y, Chen L, Feng Y, Zhou Y, Zhai Y, Lu J (2017). Meta-analysis of the safety of voriconazole in definitive, empirical, and prophylactic therapies for invasive fungal infections. BMC Infect Dis.

[68] Barbarino JM, Owusu Obeng A, Klein TE, Altman RB (2017). PharmGKB summary: voriconazole pathway, pharmacokinetics. Pharmacogenet Genomics.

[69] Jiang L, Liang S-C, Wang C, Ge G-B, Huo X-K, Qi X-Y, Deng S, Liu KX, Ma XC (2015). Identifying and applying a highly selective probe to simultaneously determine the O-glucuronidation activity of human UGT1A3 and UGT1A4. Sci Rep.

[70] Kerdpin O, Mackenzie PI, Bowalgaha K, Finel M, Miners JO (2009). Influence of N-terminal domain histidine and proline residues on the substrate Selectivities of human UDP-glucuronosyltransferase 1A1, 1A6, 1A9, 2B7, and 2B10. Drug Metab Dispos.

[71] Smith AD, Page BDG, Collier AC, Coughtrie MWH (2020). Homology modeling of human Uridine-5′-diphosphate-glucuronosyltransferase 1A6 reveals insights into factors influencing substrate and Cosubstrate binding. ACS Omega.

[72] Nair PC, Chau N, McKinnon RA, Miners JO (2020). Arginine-259 of UGT2B7 confers UDP-sugar selectivity. Mol Pharmacol.

[73] Brammer KW, Coakley AJ, Jezequel SG, Tarbit MH (1991). The disposition and metabolism of [14C] fluconazole in humans. Drug Metab Dispos Biol Fate Chem..

[74] Egunsola O, Adefurin A, Fakis A, Jacqz-Aigrain E, Choonara I, Sammons H (2013). Safety of fluconazole in paediatrics: a systematic review. Eur J Clin Pharmacol.

[75] McDonald GB, Hinds MS, Fisher LD, Schoch HG, Wolford JL, Banaji M, Hardin BJ, Shulman HM, Clift RA (1993). Veno-occlusive disease of the liver and multiorgan failure after bone marrow transplantation: a cohort study of 355 patients. Ann Intern Med.

[76] McDonald GB, Evans AT, McCune JS, Schoch G, Ostrow JD, Gooley TA (2016). Mortality outcomes after busulfan-containing conditioning treatment and haemopoietic cell transplantation in patients with Gilbert’s syndrome: a retrospective cohort study. Lancet Haematol.

[77] Gil J, Sąsiadek MM (2012). Gilbert syndrome: the UGT1A1*28 promoter polymorphism as a biomarker of multifactorial diseases and drug metabolism. Biomark Med.

[78] Sinnott-Armstrong N, Tanigawa Y, Amar D, Mars N, Benner C, Aguirre M, Venkataraman GR, Wainberg M, Ollila HM, Kiiskinen T, Havulinna AS, Pirruccello JP, Qian J, Shcherbina A, Rodriguez F, Assimes TL, Agarwala V, Tibshirani R, Hastie T, Ripatti S, Pritchard JK, Daly MJ, Rivas MA, FinnGen (2021). Genetics of 35 blood and urine biomarkers in the UK biobank. Nat Genet.

[79] Sali A, Potterton L, Yuan F, van Vlijmen H, Karplus M (1995). Evaluation of comparative protein modeling by MODELLER. Proteins..

[80] Abraham MJ, Murtola T, Schulz R, Páll S, Smith JC, Hess B, Lindahl E (2015). GROMACS: high performance molecular simulations through multi-level parallelism from laptops to supercomputers. SoftwareX..

[81] Creegan T, Jacob L, Singh R, Zhang JG (2018). Development of an in-vitro method as a tool to assess UDP-glucuronyltransferase (UGT) 2B10 inhibition. Drug Metab Pharmacokinet.

[82] Milani N, Qiu N, Molitor B, Badée J, Cruciani G, Fowler S (2020). Use of phenotypically poor metabolizer individual donor human liver Microsomes to identify selective substrates of UGT2B10. Drug Metab Dispos Biol Fate Chem..

[83] The UniProt Consortium (2021). UniProt: the universal protein knowledgebase in 2021. Nucleic Acids Res.

[84] Saxena A, Sangwan R, Mishra S (2013). Fundamentals of homology modeling steps and comparison among important bioinformatics tools: an overview. Sci Int.

[85] Blum M, Chang H-Y, Chuguransky S, Grego T, Kandasaamy S, Mitchell A, Nuka G, Paysan-Lafosse T, Qureshi M, Raj S, Richardson L, Salazar GA, Williams L, Bork P, Bridge A, Gough J, Haft DH, Letunic I, Marchler-Bauer A, Mi H, Natale DA, Necci M, Orengo CA, Pandurangan AP, Rivoire C, Sigrist CJA, Sillitoe I, Thanki N, Thomas PD, Tosatto SCE, Wu CH, Bateman A, Finn RD (2021). The InterPro protein families and domains database: 20 years on. Nucleic Acids Res.

[86] Larsson P, Wallner B, Lindahl E, Elofsson A (2008). Using multiple templates to improve quality of homology models in automated homology modeling. Protein Sci Publ Protein Soc.

[87] Pettersen EF, Goddard TD, Huang CC, Couch GS, Greenblatt DM, Meng EC, Ferrin TE (2004). UCSF chimera--a visualization system for exploratory research and analysis. J Comput Chem.

[88] Jumper J, Evans R, Pritzel A, Green T, Figurnov M, Ronneberger O, Tunyasuvunakool K, Bates R, Žídek A, Potapenko A, Bridgland A, Meyer C, Kohl SAA, Ballard AJ, Cowie A, Romera-Paredes B, Nikolov S, Jain R, Adler J, Back T, Petersen S, Reiman D, Clancy E, Zielinski M, Steinegger M, Pacholska M, Berghammer T, Bodenstein S, Silver D, Vinyals O, Senior AW, Kavukcuoglu K, Kohli P, Hassabis D (2021). Highly accurate protein structure prediction with AlphaFold. Nature..

[89] SAVES - Ramachandran Plot. http://services.mbi.ucla.edu/SAVES/Ramachandran/. Accessed 23 Nov 2020.

[90] Zhang Z, Li Y, Lin B, Schroeder M, Huang B (2011). Identification of cavities on protein surface using multiple computational approaches for drug binding site prediction. Bioinformatics..

[91] The PyMOL Molecular Graphics System. Version 2.4.1. Schrödinger, LLC.

[92] Dallakyan S, Olson AJ (2015). Small-molecule library screening by docking with PyRx. Methods Mol Biol Clifton NJ.

[93] Sadowski J, Gasteiger J, Klebe G (1994). Comparison of automatic three-dimensional model builders using 639 X-ray structures. J Chem Inf Comput Sci.

[94] Morris GM, Huey R, Lindstrom W, Sanner MF, Belew RK, Goodsell DS, Olson AJ (2009). AutoDock4 and AutoDockTools4: automated docking with selective receptor flexibility. J Comput Chem.

[95] Du X, Li Y, Xia Y-L, Ai S-M, Liang J, Sang P, et al. Insights into protein–ligand interactions: mechanisms, models, and methods. Int J Mol Sci. 2016;17(2). 10.3390/ijms17020144. 10.3390/ijms17020144 PMC4783878 26821017

[96] Lemkul J (2018). From Proteins to Perturbed Hamiltonians: A Suite of Tutorials for the GROMACS-2018 Molecular Simulation Package [Article v1.0]. Living J Comput Mol Sci.

[97] Oostenbrink C, Villa A, Mark AE, van Gunsteren WF (2004). A biomolecular force field based on the free enthalpy of hydration and solvation: the GROMOS force-field parameter sets 53A5 and 53A6. J Comput Chem.

[98] Schüttelkopf AW, van Aalten DMF (2004). PRODRG: a tool for high-throughput crystallography of protein–ligand complexes. Acta Crystallogr D Biol Crystallogr.

[99] Kumari R, Kumar R, Lynn A (2014). g_mmpbsa—a GROMACS tool for high-throughput MM-PBSA calculations. J Chem Inf Model.

