# Correction to: A potential implication of UDP-glucuronosyltransferase 2B10 in the detoxification of drugs used in pediatric hematopoietic stem cell transplantation setting: an in silico investigation

**DOI:** 10.1186/s12860-022-00407-8

**Published:** 2022-01-27

**Authors:** Shannon Robin, Khalil Ben Hassine, Jayaraman Muthukumaran, Simona Jurkovic Mlakar, Maja Krajinovic, Tiago Nava, Chakradhara Rao S. Uppugunduri, Marc Ansari

**Affiliations:** 1 CANSEARCH Research Platform in Pediatric Oncology and Hematology, Department of Pediatrics, Gynecology and Obstetrics, University of Geneva, Geneva, Switzerland; 2 Department of Biotechnology, School of Engineering and Technology, Sharda University, Greater Noida, 201306 India; 3 Charles-Bruneau Cancer Center, CHU Sainte-Justine Research Center, Departments of Pediatrics and Pharmacology, University of Montreal, Montreal, Quebec Canada; 4 Division of Pediatric Oncology and Hematology, Department of Women, Child and Adolescent, University Geneva Hospitals, Geneva, Switzerland

**Correction to: BMC Mol Cell Biol 23, 5 (2022)**

**https://doi.org/10.1186/s12860-021-00402-5**

Following publication of the original article [1], the following typesetting error was noticed:

1. The equal contribution note has been updated.
2. Table 1, column 4 (InterPro-Protein Family), row 3 “ransferase” should be “transferase”
3. The footnote for Table 3 was mistakenly added to the body text (last para in pg 10 [1], continued as para 1 in pg.12 i.e. “Results are presented as the mean±SD………....to compare our results with other putative ligands” The correct Table 3 and footnote are supplied below.

**Table 3:** Estimated binding free energy and dissociation constant between putative substrates and human UGT2B10

Model	Substrate	Ligand	***ΔG*** [Kcal/mol ± SD]	Kd [mM]
**UGT2B10 with UDPGlcA**	**Controls**	**Amitriptyline**	**−1.9 ± 0.2**	**39.0**
		**Itraconazole**	19.0 ± 0.5	1.1 × 10^17^
	**Putative ligands**	**4-hydroxy voriconazole**	**−1.0 ± 0.0**	**184.7**
		**Acetaminophen**	**−5.5 ± 0.0**	**0.1**
		Cyclosporine A	154.9 ± 2.9	1.8 × 10^118^
		Bilirubine	6.9 **± 0.0**	1.2 × 10^15^
		Dihydroxy voriconazole	−0.6 ± 0.0	363.0
		**Hydroxy voriconazole**	**−1.2 ± 0.1**	**125.0**
		**Lorazepam**	**−2.6 ± 0.0**	**12.4**
		Methotrexate	−0.5 ± 0.5	567.3
		Methylprednisolone	5.2 ± 0.1	6.2 × 10^6^
		**Mycophenolic acid**	**−5.1 ± 0.1**	**0.2**
		Posaconazole	17.6 ± 0.3	8.8 × 10^15^
		UDCA-G1	2.2 ± 0.1	4.4 × 10^4^
		UDCA-G2	1.2 ± 0.1	8053.6
		Ursodeoxycholic acid	2.2 ± 0.1	4.4 × 10^4^
		**Voriconazole**	**−1.0 ± 0.1**	**197.8**
		**Voriconazole N-oxide**	**−2.3 ± 0.1**	**2.1 × 10** ^**4**^
		**Voriconazole N-oxide intermediate UK-215,364** [35]	**−6.4 ± 0.1**	**0.02**

Results are presented as the mean ± SD of three different replicates. *Kd* dissociation constant, *UDCA-G1 and UDCA-G2* ursodeoxycholic acid glucuronide conjugate 1 and 2 [44], *UDPGlcA* UDP-glucuronic acid. Molecules with ΔG of < −0.1 and with an SD of ≤0.1 Kcal/mol were selected for further for MD simulations (methotrexate was not selected as it has an SD 0.5). SD is calculated from 8 docking poses or models (default option). The ligand binding pose was selected for further analyses is the pose with the lowest free binding energy (Kcal/mol). Bilirubin was selected for further molecular docking simulations as an endogenous negative control to compare our results with other putative ligands

4) The footnote for Table 5 was mistakenly added to the body text. The correct Table 5 and footnote are supplied below.

**Table 5:** Average values of hydrogen bonds, RMSD, RMSF, RoG, SASA, trace of the covariance matrix values and MM/PBSA binding free energy values of the different UGT2B10 with putative substrates

Complex	Average number of intra-molecular hydrogen bonds ± SD	Average number of inter-molecular hydrogen bonds ± SD	Average RMSD [nm ± SD]	Average RoG [nm]	Average RMSF [nm ± SD]	Average SASA [nm^**2**^ ± SD]	Trace of the covariance matrix [nm^**2**^]	MMPBSAbinding free energy [kcal/mol]
UGT2B10 apo form	302.73 ± 9.60	N/A	0.39 ± 0.05	2.26 ± 1.38*10^− 2^	0.20 ± 0.08	216.39 ± 4.74	42.08	NA
UGT2B10-UDPGlcA	305.29 ± 11.70	7.54 ± 2.01	0.43 ± 0.05	2.28 ± 9.57*10^− 4^	0.19 ± 0.09	221.01 ± 5.74	38.82	NC
UGT2B10-UDPGlcA-AMT	290.12 ± 10.25	0.21 ± 0.41	0.49 ± 0.05	2.29 ± 1.16*10^− 2^	0.21 ± 0.12	227.45 ± 4.47	55.6	− 160.85 ± 10.99
UGT2B10-UDPGlcA-APAP	304.67 ± 8.96	1.16 ± 0.68	0.47 ± 0.08	2.24 ± 2.84*10^− 2^	0.25 ± 0.11	224.61 ± 4.02	76.97	− 174.24 ± 13.38
UGT2B10-UDPGlcA-BIL	292.97 ± 11.69	0.00 ± 0.00	0.39 ± 0.07	2.31 ± 1.88*10^− 2^	0.21 ± 0.13	234.64 ± 3.93	65.10	−104.00 ± 11.06
UGT2B10-UDPGlcA-ITZ	310.11 ± 10.43	0.24 ± 0.44	0.51 ± 0.05	2.33 ± 1.29*10^− 2^	0.23 ± 0.14	244.84 ± 6.86	60.70	− 127.79 ± 15.25
UGT2B10-UDPGlcA-LOR	300.25 ± 8.69	0.65 ± 0.79	0.42 ± 0.06	2.28 ± 1.14*10^− 2^	0.20 ± 0.10	230.58 ± 3.67	48.81	− 162.07 ± 19.30
UGT2B10- UDPGlcA-MPA	303.7 ± 9.09	2.48 ± 1.33	0.47 ± 0.05	2.33 ± 1.21*10^− 2^	0.22 ± 0.16	230.48 ± 6.97	60.23	− 158.46 ± 11.95
UGT2B10-UDPGlcA-VCZ	301.51 ± 9.69	0.76 ± 0.96	0.49 ± 0.07	2.31 ± 2.16*10^− 2^	0.24 ± 0.16	234.23 ± 5.28	87.61	−59.56 ± 17.13
UGT2B10-UDPGlcA-HVCZ	311.18 ± 9.89	0.87 ± 0.66	0.46 ± 0.05	2.27 ± 1.13*10^− 2^	0.19 ± 0.09	224.92 ± 5.18	40.50	−85.43 ± 14.23
UGT2B10-UDPGlcA-DHVCZ	308.33 ± 9.97	1.21 ± 0.83	0.43 ± 0.04	2.29 ± 1.13*10^− 2^	0.20 ± 0.10	227.99 ± 4.71	43.27	−86.34 ± 25.35
UGT2B10-UDPGlcA-4HVCZ	309.36 ± 11.59	0.00 ± 0.00	0.44 ± 0.03	2.26 ± 8.81*10^− 3^	0.18 ± 0.09	223.49 ± 6.05	38.55	−95.43 ± 18.47
UGT2B10-UDPGlcA-VCZ-N-O	303.01 ± 10.28	1.38 ± 0.78	0.43 ± 0.05	2.30 ± 1.28*10^− 2^	0.22 ± 0.11	233.91 ± 5.20	58.16	− 12.41 ± 19.02
UGT2B10-UDPGlcA-VCZ-N-O-intermediate UK-215,364 [35]	305.54 ± 12.28	0.72 ± 0.52	0.38 ± 0.03	2.3 ± 1.19*10^− 2^	0.18 ± 0.1	227.28 ± 4.55	37.75	− 164.44 ± 13.38

Results are indicated as mean ± SD of the MD simulation’s analysis results. *4HVCZ* 4-hydroxy voriconazole, *AMT* amitriptyline, *APAP* acetaminophen, *BIL* bilirubin, *DHVCZ* Di-hydroxy voriconazole, *HVCZ* Hydroxy voriconazole, *ITZ* itraconazole, *LOR* lorazepam, *MPA* mycophenolic acid, *NC* Not calculated, *VCZ-N-O* voriconazole N-oxide, *RMSD* root mean square deviation, *RMSF* root mean square fluctuation, *RoG* radius of gyration, *UDPGlcA* UDP-glucuronic acid

5) In the section *Molecular dynamics simulations with GROMACS* “Eight compounds showing ΔG value of ≤ −1.0 kcal/mol predicted by AutoDock Vina”, should be “Eight compounds showing ΔG value of ≤ −0.1 kcal/mol predicted by AutoDock Vina”
