# Supplementary material for: A potential implication of UDP-glucuronosyltransferase 2B10 in the detoxification of drugs used in pediatric hematopoietic stem cell transplantation setting: an in silico investigation
Source: BMC Mol Cell Biol. 2022 Jan 21;23:5. doi: 10.1186/s12860-021-00402-5 (PMC8781437; doi:10.1186/s12860-021-00402-5)
Supplement: Supplementary file 1 — Additional file 1. List of in vitro and in vivo UGT2B10 drug ligands. IC50: half inhibitory concentration; Ki: inhibitory constant; Km: Michaelis constant; N/A: not applicable. [file 12860_2021_402_MOESM1_ESM.docx]

Additional file 1. List of *in vitro* and *in vivo* UGT2B10 drug ligands. IC50: half inhibitory concentration; *Ki*: inhibitory constant; Km: Michaelis-Menten constant; N/A: not applicable

| **Substrate** | **Level of evidence** | **Usage** | **Chemical class** | **Affinity** | **Source** |
| --- | --- | --- | --- | --- | --- |
| Amitriptyline | *In vitro* | Tricylic antidepressant | Tertiary aliphatic amine | IC50 = 6.45 ± 0.46 µM ^1^ | ^1,2^ |
| Asenapine | *In vitro* | Antipsychotic | Tertiary amine | N/A | ^2^ |
| Chlorcyclizine | *In vitro* | Antihistamine | Tertiary amine | N/A | ^2^ |
| Chlorpromazine | *In vitro* | Antipsychotic | Tertiary aromatic amine | N/A | ^2^ |
| Clomipramine | *In vitro* | Tricylic antidepressant | Tertiary aromatic amine | IC50 = 26 ± 0.49 µM | ^1^ |
| Clozapine | *In vitro* | Antipsychotic | Tertiary amine | IC50 = 61.3 ± 1.4 µM ^1^ | ^1,2^ |
| Cotinine | *In vivo* | Tobacco products | Tertiary amine | Km = 0.93 - 5.43 mM | ^1^ |
| Cyclizine | *In vitro* | Antihistamine | Tertiary amine | N/A | ^2^ |
| Cyclobenzaprine | *In vitro* | Muscle relaxer | Tertiary aliphatic amine | N/A | ^2^ |
| Desloratadine | *In vitro* | Antihistamine | Secondary aliphatic amine | Ki = 1.3 µM ^3^  IC50 = 3.86 ± 0.05 µM ^1^ | ^1,3^ |
| Diphenhydramine | *In vitro* | Antihistamine | Tertiary aliphatic amine | N/A | ^4^ |
| Dothiepin | *In vitro* | Antidepressant | Tertiary aliphatic amine | N/A | ^2^ |
| Doxepin | *In vitro* | Antidepressant | Tertiary aliphatic amine | IC50 = 3.64 ± 0.16 µM ^1^ Ki = 0.95 µM | ^1,2^ |
| Fluconazole | *In vitro* | Antifungal | Triazole | Inhibitor IC50 = 1136 ± 88.4 µM | ^1^ |
| Imatinib | *In vitro* | Tyrosine kinase inhibitors | Tertiary amine | N/A | ^2^ |
| Imipramine | *In vitro* | Tricylic antidepressant | Tertiary aliphatic amine | IC50 = 42.8 ± 1.52 µM ^1^  Ki = 380 ± 250 µM ^4^ | ^1,4^ |
| Loxapine | *In vitro* | Antipsychotic | Tertiary amine | N/A | ^2^ |
| Ketoconazole | *In vitro* | Antifungal | Tertiary aliphatic amine | IC50 = 11.9 ± 1.69 µM ^1^ | ^1,4^ |
| Ketotifen | *In vitro* | Antihistamine | Tertiary amine | N/A | ^4^ |
| Loratadine | *In vitro* | Antihistamine | Tertiary amine | IC50 = 2.18 ± 0.34 µM | ^1^ |
| Mianserin | *In vitro* | Tetracyclic antidepressant | Tertiary amine | IC50 = 2.24 ± 0.11 µM Ki = 0.43 µM | ^1,2^ |
| Midazolam | *In vitro* | Benzodiazepine | Azole | N/A | ^4^ |
| Mirtazapine | *In vitro* | Antidepressant | Tertiary aliphatic amine | IC50 = 31 ± 0.99 µM ^1^ | ^1,2^ |
| Nicotine | *In vivo* | Tobacco products | Tertiary amine | Km = 0.29 mM ^5^ | ^5^ |
| Niflumic acid | *In vitro* | Nonsteroidal anti-inflammatory | Secondary amine | IC50 = 168 ± 0.14 µM | ^1^ |
| Olanzapine | *In vitro* | Bipolar trouble, schizophrenia | Tertiary aliphamic amine | IC50 = 276 ± 4.49 µM ^1^ | ^1,4^ |
| Phenylbutazone | *In vitro* | Nonsteroidal anti-inflammatory | Secondary amine | IC50 = 220 ± 35.4 µM | ^1^ |
| Pizotifen | *In vitro* | Reducing migraines | Tertiary aliphatic amine | N/A | ^4^ |
| Promethazine | *In vitro* | Antihistamine | Amine | N/A | ^2^ |
| Tamoxifen | *In vitro* | A modulator of estrogen receptors | Tertiary amine | N/A | ^4^ |
| Trimipramine | *In vitro* | Tricylic antidepressant | Tertiary aliphatic amine | IC50 = 32.6 ± 1.7 µM | ^1^ |
