# Supplementary material for: A potential implication of UDP-glucuronosyltransferase 2B10 in the detoxification of drugs used in pediatric hematopoietic stem cell transplantation setting: an in silico investigation
Source: BMC Mol Cell Biol. 2022 Jan 21;23:5. doi: 10.1186/s12860-021-00402-5 (PMC8781437; doi:10.1186/s12860-021-00402-5)
Supplement: Supplementary file 2 — Additional file 2. Quality of the final UGT2B10 and AlphaFold model, based on ERRAT, Verify3D, ProSA and Ramachandran plots. Comparison with AlphaFold structure is also given in the table. [file 12860_2021_402_MOESM2_ESM.docx]

Additional file 2. Quality of the final UGT2B10 and AlphaFold model, based on ERRAT, Verify3D, ProSA and Ramachandran plots. Comparison with AlphaFold structure is also given in the table.

| **Model** | **ERRAT**  (Threshold for a good model > 90%) | **Verify3D**  (Threshold for a good model > 80%) | **ProSA** | **Ramachandran Plot**  **Favoured and allowed region**  (Threshold for a good model > 90%) | **Ramachandran Plot**  **Generously allowed and disallowed region**  (Must be closer to 0% as possible) |
| --- | --- | --- | --- | --- | --- |
| Initial | 45.6% | 76.9% | -7.21 | 89.3% | 2.8% |
| GalaxyRefine1 | 70.7% | 79.5% | -8.3 | 90.8% | 1.7% |
| GalaxyRefine2 | 72.2% | 78.6% | -8.5 | 91.9% | 1.7% |
| GalaxyRefine3 | 72.6% | 74.8% | -8.7 | 93.2% | 1.5% |
| GalaxyRefine3  Yasara1 | 88% | 74.1% | -9.02 | 91.9% | 1.5% |
| GalaxyRefine3  Yasara2 | 91.4% | 71.5% | -9.08 | 92.6% | 1.5% |
| GalaxyRefine3  Yasara3  (Final model) | 93.3% | 74.8% | -8.78 | 92.3% | 1.7% |
| Secondary Structural Composition of our model (PDBSum) | 3 sheets, 6 beta alpha beta units, 1 beta bulge, 13 strands, 27 helices, 23 helix-helix interactions, 34 beta turns, and 11 gamma turns | | | | |
| AlphaFold | 93.9% | 95.65% | -11.58 | 100% | 0% |
| Secondary Structural Composition of AlphaFold model (PDBSum) | 2 sheets, 6 beta alpha beta units, 1 beta bulge, 13 strands, 21 helices, 23 helix-helix interactions, 30 beta turns, and 6 gamma turns | | | | |
| RMSD between our model and AlphaFold model (Å) | 2.98 | | | | |
