# Supplementary figures and images for: A potential implication of UDP-glucuronosyltransferase 2B10 in the detoxification of drugs used in pediatric hematopoietic stem cell transplantation setting: an in silico investigation
Source: BMC Mol Cell Biol. 2022 Jan 21;23:5. doi: 10.1186/s12860-021-00402-5 (PMC8781437; doi:10.1186/s12860-021-00402-5)

Additional file 3. Secondary structure of UGT2B10 obtained with PDBSum.


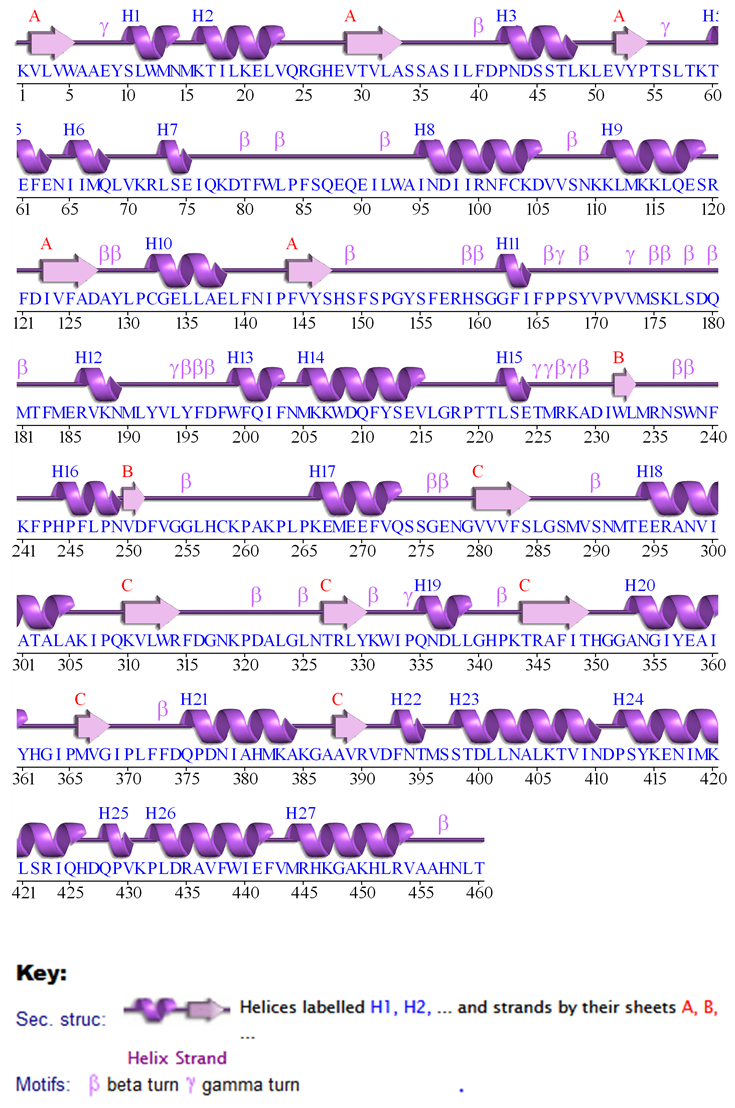

Supplement: Supplementary file 3 — Additional file 3. Secondary structure of UGT2B10 obtained with PDBsum. [file 12860_2021_402_MOESM3_ESM.docx]

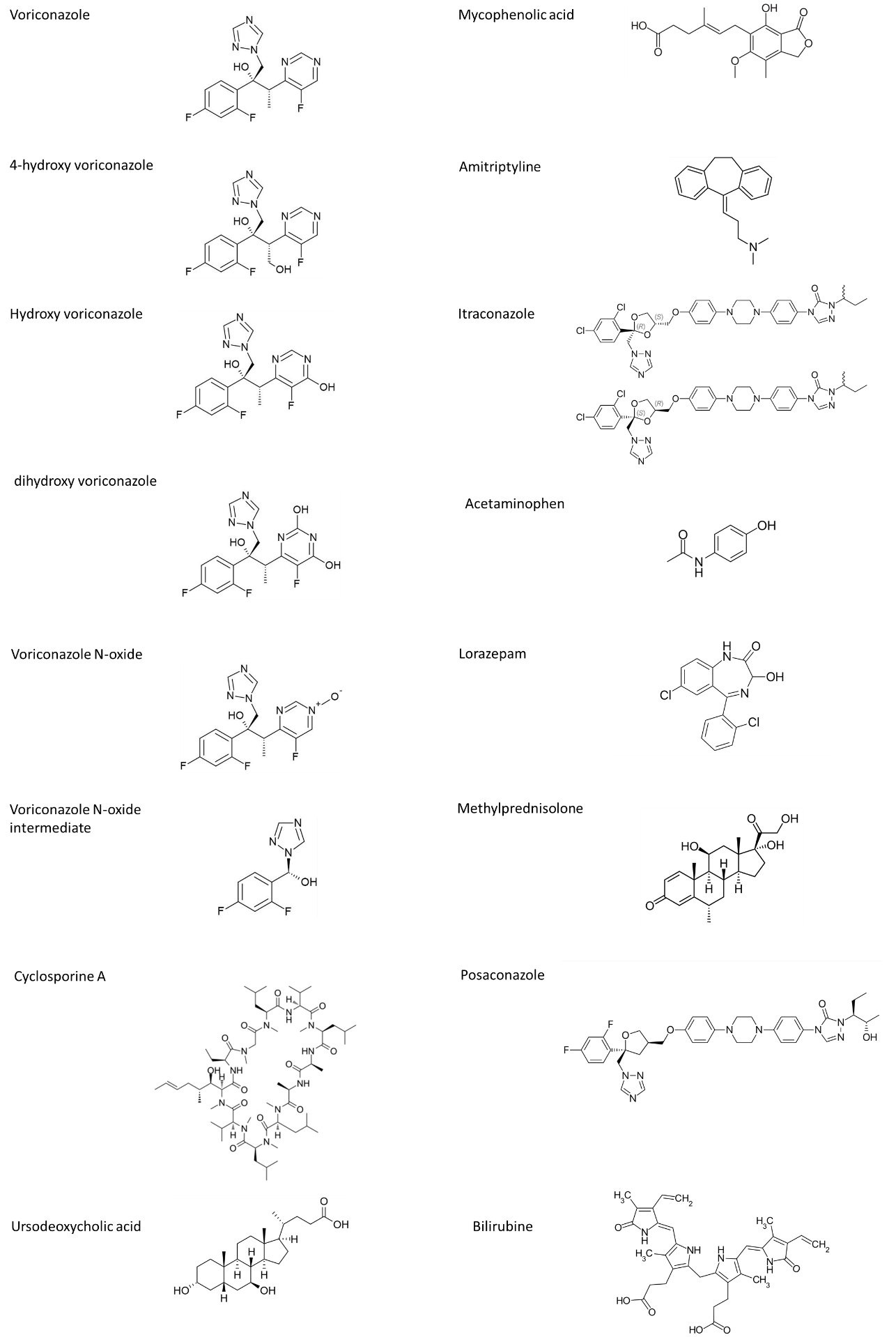


Additional file 5: Structure of the selected molecules to perform the molecular docking.

Supplement: Supplementary file 5 — Additional file 5. Structures of the selected molecules to perform the molecular docking. [file 12860_2021_402_MOESM5_ESM.docx]
