# Supplementary material for: A potential implication of UDP-glucuronosyltransferase 2B10 in the detoxification of drugs used in pediatric hematopoietic stem cell transplantation setting: an in silico investigation
Source: BMC Mol Cell Biol. 2022 Jan 21;23:5. doi: 10.1186/s12860-021-00402-5 (PMC8781437; doi:10.1186/s12860-021-00402-5)
Supplement: Supplementary file 4 — Additional file 4. Predicted A) cofactor binding site, B) substrate-binding site of human UGT2B10 model (The green box represents the grid box for the ligand docking simulation with AutoDock Vina), C) Structural superposition analysis of our model with the AlphaFold Model (Display: PyMOL) Red: AlphaFold; Blue: Our Model. [file 12860_2021_402_MOESM4_ESM.docx]

| A | 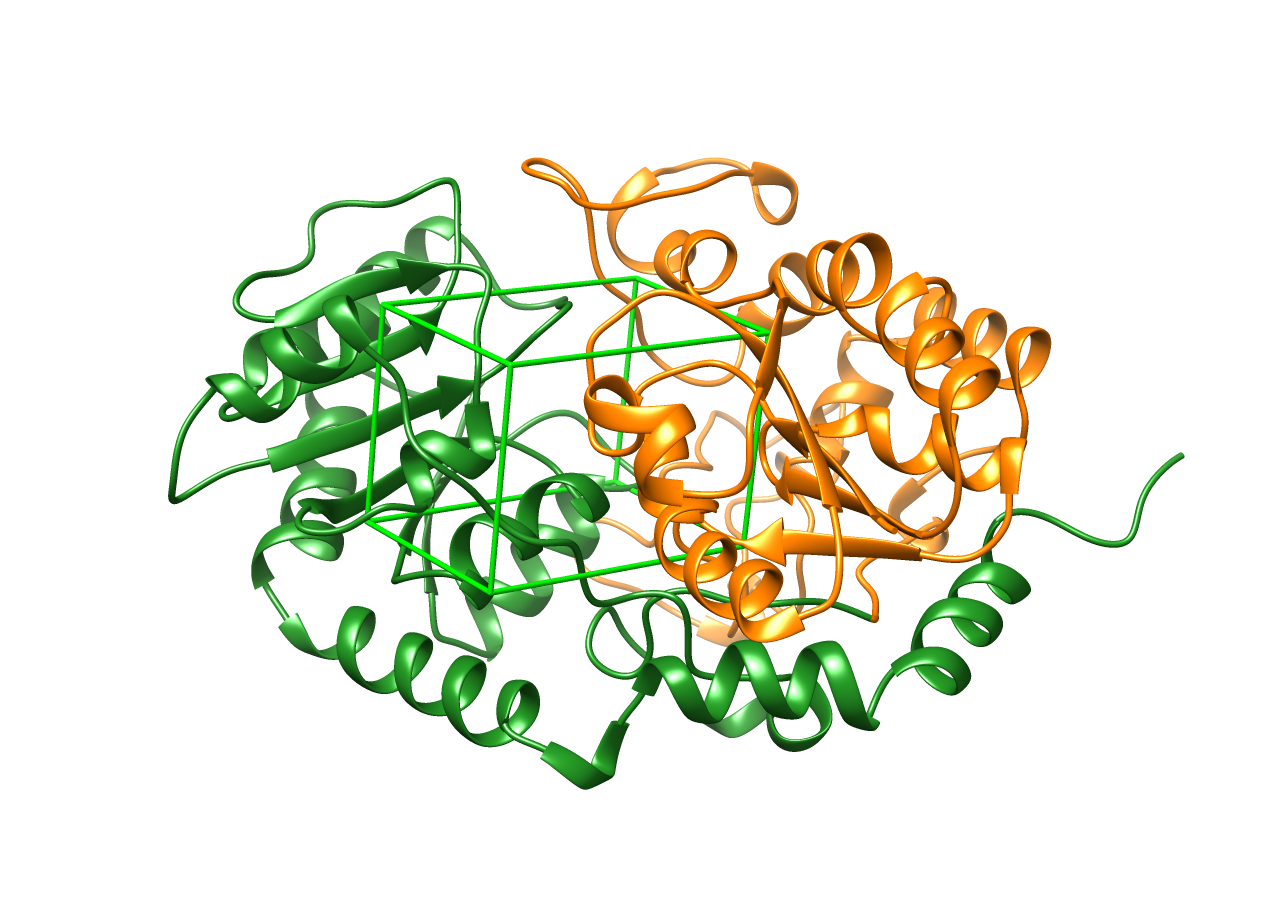 |
| --- | --- |
| B | 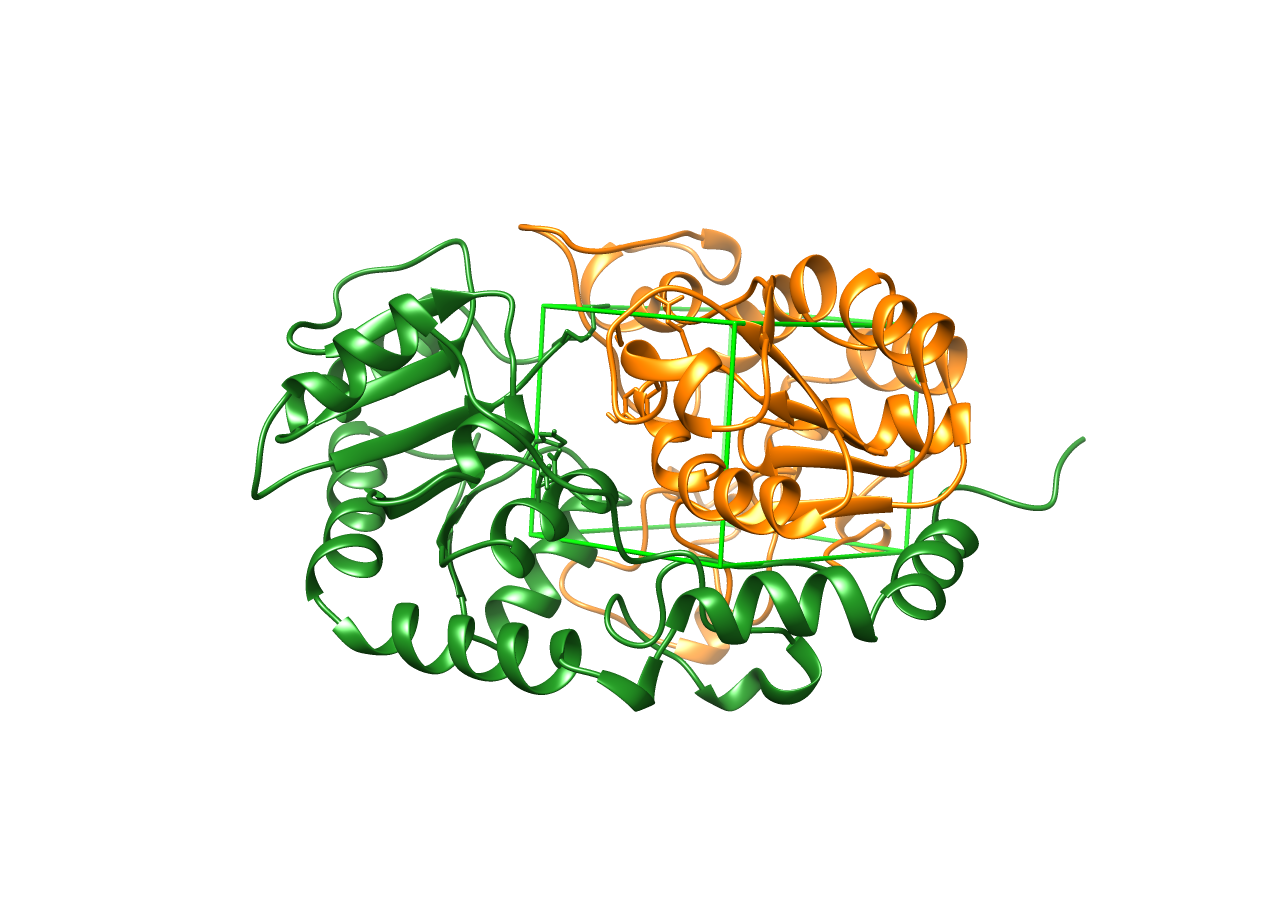 |
|  |  |

C


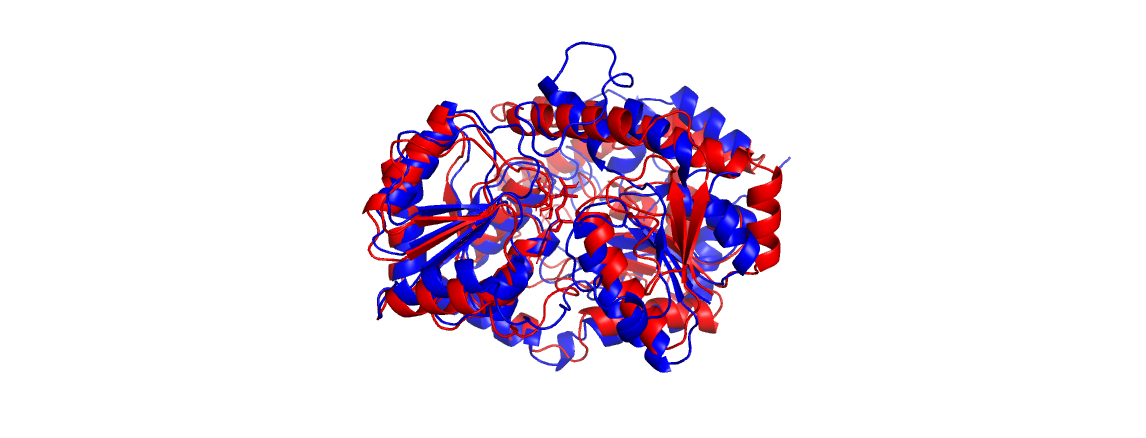


Additional file 4. Predicted A) cofactor binding site, B) substrate-binding site of human UGT2B10 model (The green box represents the grid box for the ligand docking simulation with AutoDock Vina), C) Structural superposition analysis of our model with the AlphaFold Model (Display: PyMOL) Red: AlphaFold; Blue: Our Model.
