# Supplementary material for: A potential implication of UDP-glucuronosyltransferase 2B10 in the detoxification of drugs used in pediatric hematopoietic stem cell transplantation setting: an in silico investigation
Source: BMC Mol Cell Biol. 2022 Jan 21;23:5. doi: 10.1186/s12860-021-00402-5 (PMC8781437; doi:10.1186/s12860-021-00402-5)
Supplement: Supplementary file 6 — Additional file 6. Comparative molecular docking results of refined model of UGTB10 and AlphaFold model with the selected ligands. [file 12860_2021_402_MOESM6_ESM.docx]

| **Type** | **Ligand** | **Homology model Free binding energy (Kcal/mol) generated by Modller** | **AlphaFold Free binding energy (Kcal/mol)** |
| --- | --- | --- | --- |
| **Positive control** | Amitriptyline | -2.0 | -8.6 |
| **Positive control** | Chlorpromazine | -2.0 | -7.8 |
| **Positive control** | Clozapine | 0.5 | -9.1 |
| **Positive control** | Cotinine | -5.3 | -6.0 |
| **Positive control** | Cyclizine | -4.6 | -8.5 |
| **Positive control** | Cyproheptadine | 5.9 | -10.3 |
| **Positive control** | Desloratadine | 4.8 | -9.7 |
| **Positive control** | Fluoxetine | -6.7 | -8.5 |
| **Positive control** | Ketoconazole | 3.3 | -9.2 |
| **Positive control** | Mianserine | -4.8 | -10.4 |
| **Positive control** | Selegiline | -6.0 | -6.3 |
| **Positive control** | Aripiprazole | -1.6 | -9.6 |
| **Positive control** | Asenapine | -2.2 | -8.6 |
| **Positive control** | Azatadine | -3.3 | -3.2 |
| **Positive control** | Chlorcyclizine | -3.4 | -8.9 |
| **Positive control** | citalopram | -1.3 | -8.8 |
| **Positive control** | clomipramine | -0.8 | -7.8 |
| **Positive control** | desmethylnortriptyline | -4.4 | -8.8 |
| **Positive control** | desvenlafaxine | -4.9 | -8.7 |
| **Positive control** | dexmedetomidine | -6 | -7.3 |
| **Positive control** | didesimipramine | -3.1 | -8.2 |
| **Positive control** | doxepine | -2.2 | -8.3 |
| **Positive control** | duloxetine | -5.8 | -8.4 |
| **Positive control** | fluphenazine | -0.4 | -8.9 |
| **Positive control** | fluvoxamine | -6.5 | -6.7 |
| **Positive control** | imipramine | -1.6 | -8.4 |
| **Positive control** | levomedetomidine | -6.3 | -7.5 |
| **Positive control** | loratadine | 8 | -8.1 |
| **Positive control** | loxapine | 0 | -8.8 |
| **Positive control** | mirtazapine | -1.8 | -10.4 |
| **Positive control** | niflumic_acid | -6.4 | -8.6 |
| **Positive control** | nortriptyline | -3.8 | -8.6 |
| **Positive control** | olanzapine | -0.5 | -8.4 |
| **Positive control** | paroxetine | -4.4 | -9 |
| **Positive control** | perphenazine | -1.5 | -8.8 |
| **Positive control** | phenelzine | -5.9 | -6.2 |
| **Positive control** | phenylbutazone | -3.7 | -8.2 |
| **Positive control** | quetiapine | 2.3 | -9.7 |
| **Positive control** | sertraline | -4.1 | -8.7 |
| **Positive control** | trimipramine | -0.6 | -8.3 |
| **Positive control** | Tamoxifen | -1.7 | -8.7 |
| **Positive control** | Nicotine | -4.7 | -5.7 |
| **Negative control** | haloperidol | -3.4 | -8.9 |
| **Negative control** | Tranylcypromine | -5.6 | -6.2 |
| **Negative control** | Venlafaxine | -3.1 | -7.5 |
| **Negative control** | Carbamazepine | -3.1 | -8.8 |
| **Negative control** | Itraconazole | 18.5 | -9.4 |
| **Negative control** | Hecogenin | 9.2 | -12.1 |

Additional File 6: Comparative molecular docking results of refined UGT2B10 and AlphaFold models with the selected ligands
