# Supplementary material for: A potential implication of UDP-glucuronosyltransferase 2B10 in the detoxification of drugs used in pediatric hematopoietic stem cell transplantation setting: an in silico investigation
Source: BMC Mol Cell Biol. 2022 Jan 21;23:5. doi: 10.1186/s12860-021-00402-5 (PMC8781437; doi:10.1186/s12860-021-00402-5)
Supplement: Supplementary file 7 — Additional file 7. Examples of electrostatic graphs of UGT2B10 with the tested ligands. A) UDPGlcA B) amitriptyline C) lorazepam. Red indicates negatively charged amino acid residues and blue positively charged amino acid residues. White indicates neutral residues. [file 12860_2021_402_MOESM7_ESM.docx]

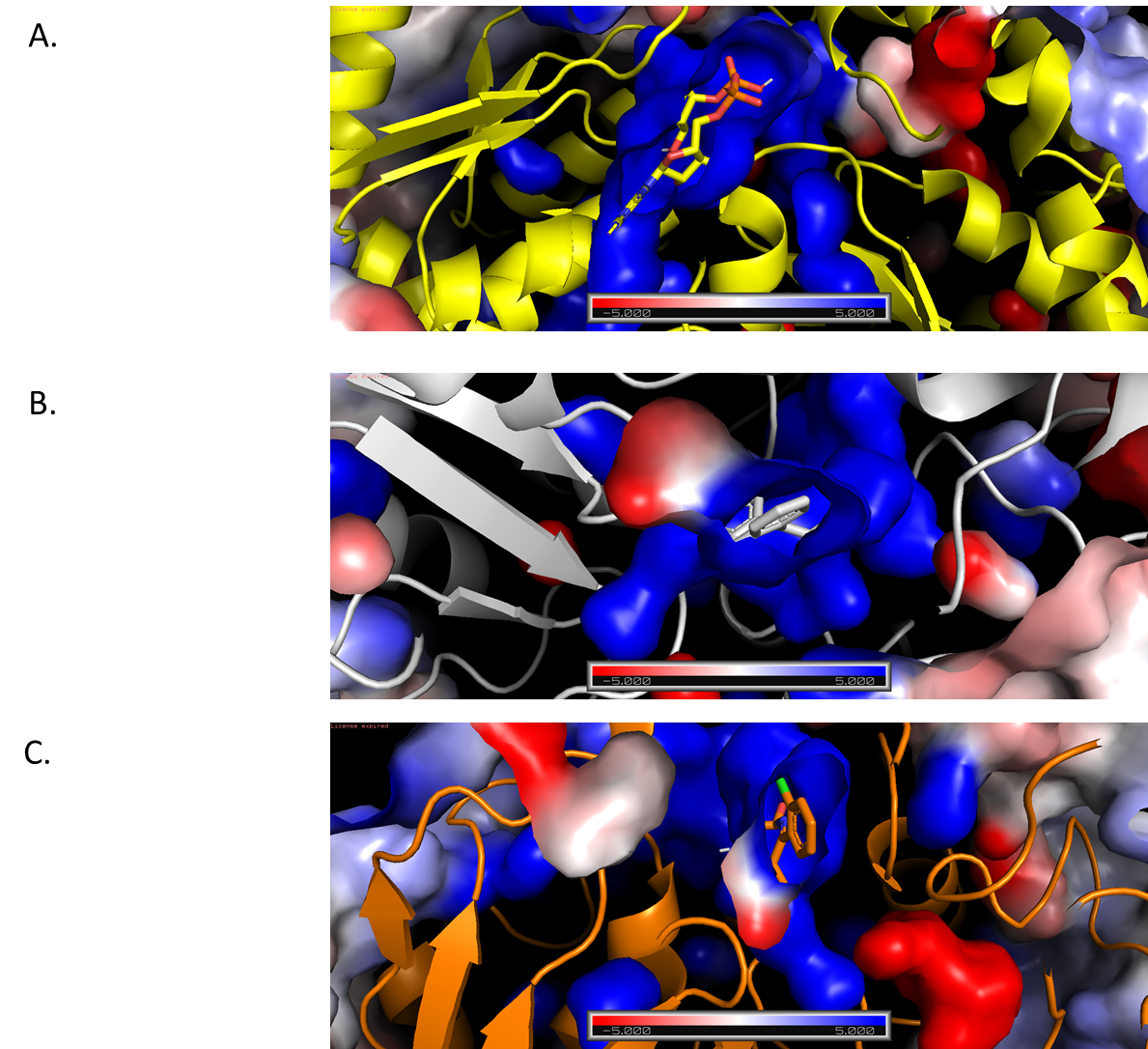


Additional file 7. Examples of electrostatic graphs of UGT2B10 binding pocket with the co-factor and tested ligands the tested ligands. A) UDPGlcA B) amitriptyline C) lorazepam. Red indicates negatively charged amino acid residues and blue positively charged amino acid residues. White indicates neutral residues.
