# Supplementary material for: A potential implication of UDP-glucuronosyltransferase 2B10 in the detoxification of drugs used in pediatric hematopoietic stem cell transplantation setting: an in silico investigation
Source: BMC Mol Cell Biol. 2022 Jan 21;23:5. doi: 10.1186/s12860-021-00402-5 (PMC8781437; doi:10.1186/s12860-021-00402-5)
Supplement: Supplementary file 8 — Additional file 8 Identification of cofactor location of UGT2B10 Model (by superposition with UGT76G from Stevia rebaudiana (PDB ID: 6INF) (Color codes: Blue: UDP orientation in UGT2B10 and Magenta: UGP orientation in crystal structure). [file 12860_2021_402_MOESM8_ESM.docx]

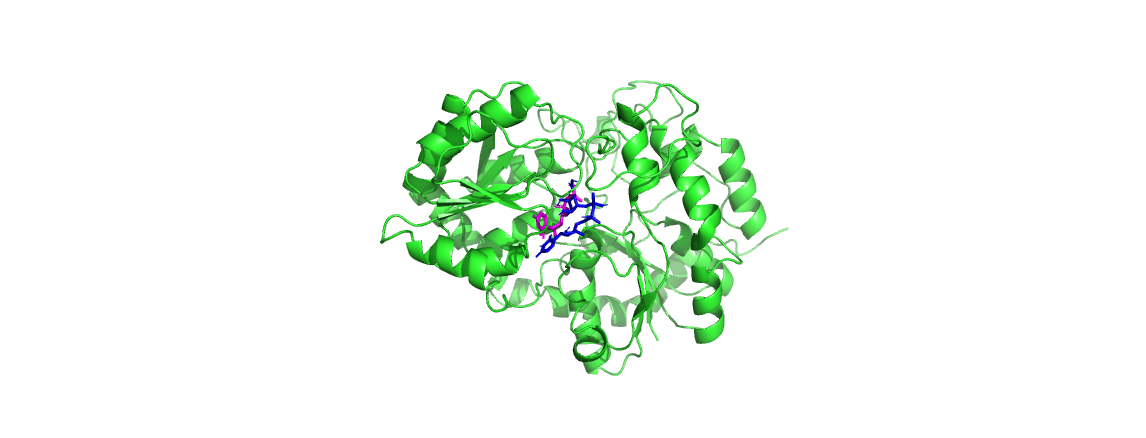


Additional file 8: Identification of cofactor location of UGT2B10 Model (by superposition with UGT76G from *Stevia rebaudiana* (PDB ID: 6INF) (Color codes: Blue: UDP orientation in UGT2B10 and Magenta: UGP orientation in crystal structure)
