# Supplementary material for: A potential implication of UDP-glucuronosyltransferase 2B10 in the detoxification of drugs used in pediatric hematopoietic stem cell transplantation setting: an in silico investigation
Source: BMC Mol Cell Biol. 2022 Jan 21;23:5. doi: 10.1186/s12860-021-00402-5 (PMC8781437; doi:10.1186/s12860-021-00402-5)
Supplement: Supplementary file 9 — Additional file 9. Domain distance analysis of UDP with various ligands (a): AMT: RED, ITZ: BLUE, BIL: YELLOW, VCZN: GREEN, VCZ: ORANGE. (b) AMT: RED, ITZ: BLUE, MPA: CYAN, APAP: MAGENTA, LORAZEPAM: DARK GREEN and (c): AMT: RED, ITZ: BLUE, DHVCZ: PURPLE, HVCZ: GOLD, 4HVCZ: OLIVE [file 12860_2021_402_MOESM9_ESM.docx]

| (a)  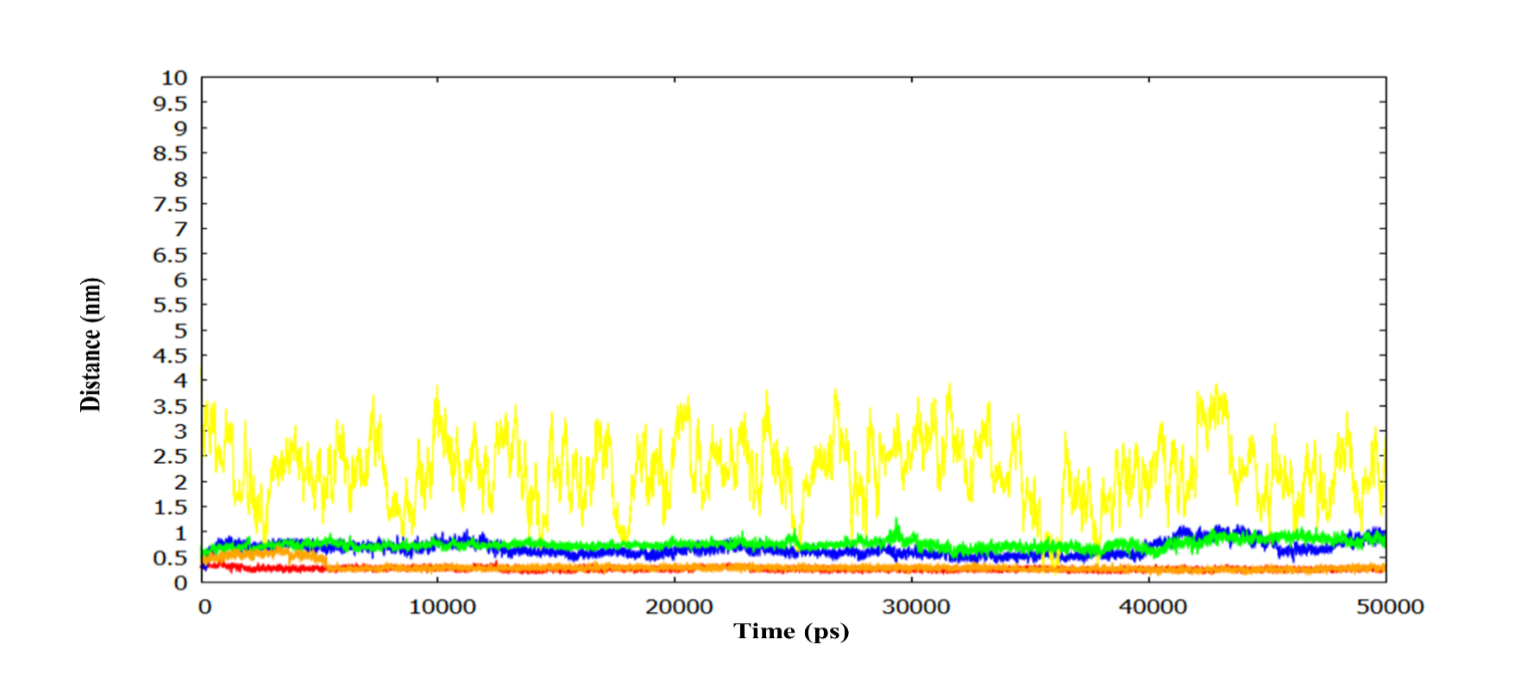  (b)  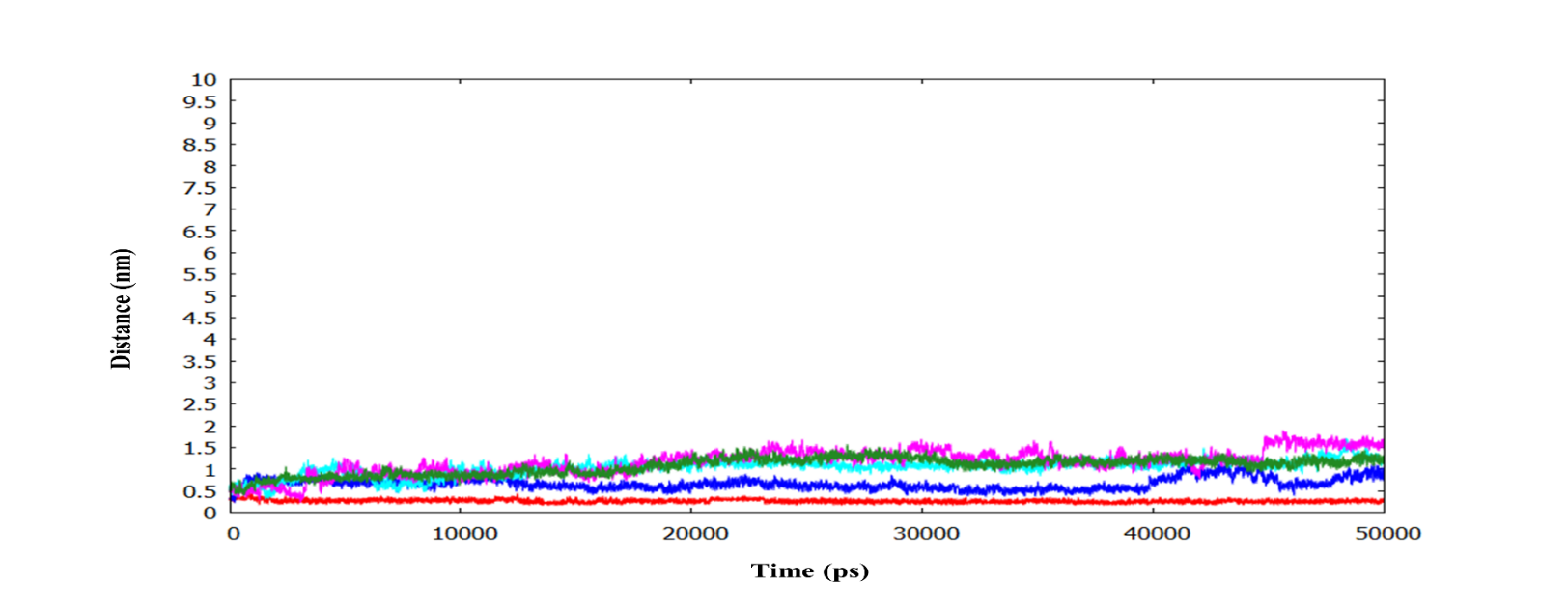  (c)  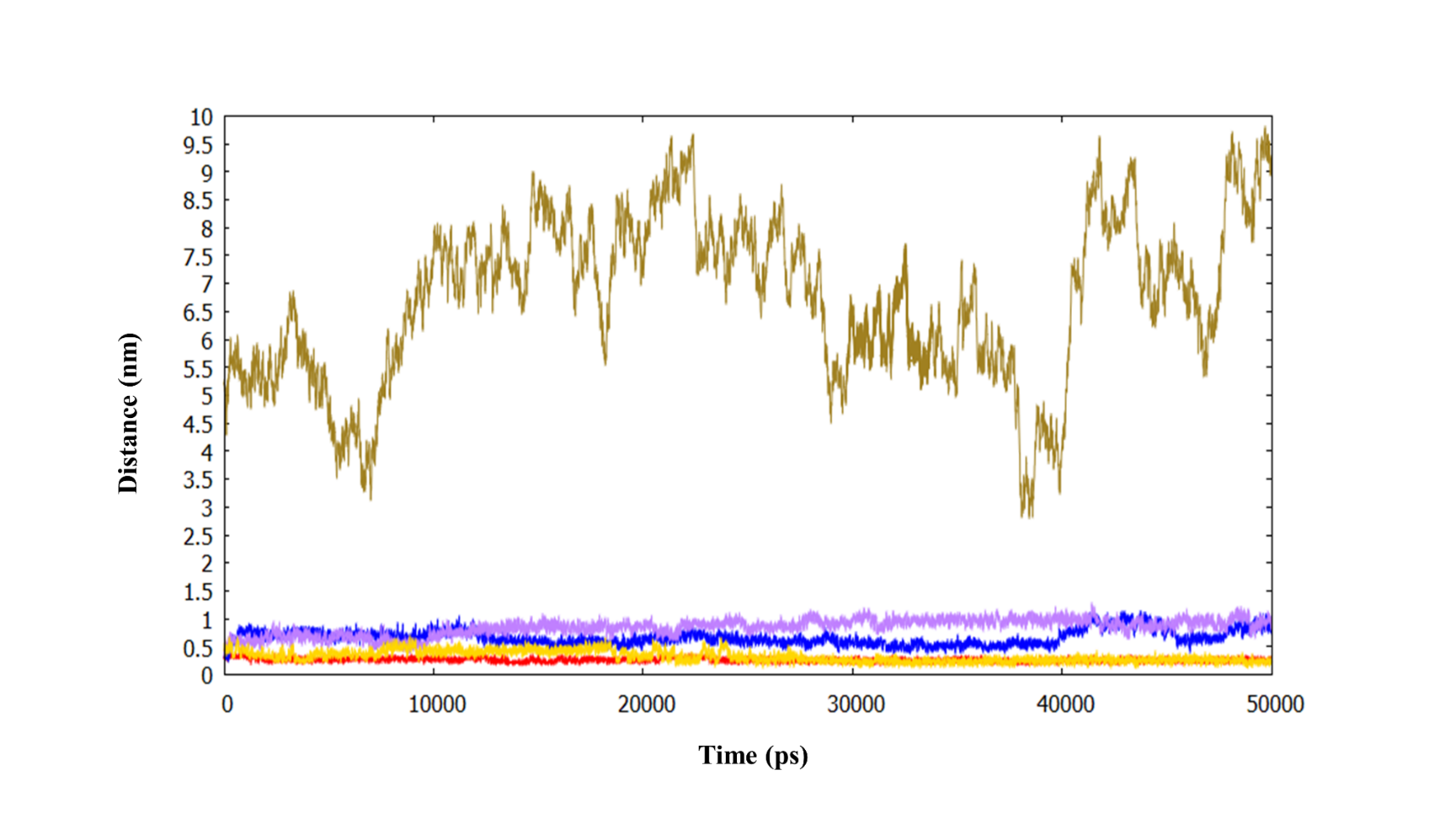 |
| --- |

Additional file 9: Domain distance analysis of UDP with various ligands (a): AMT: RED, ITZ: BLUE, BIL: YELLOW, VCZN: GREEN, VCZ: ORANGE. (b) AMT: RED, ITZ: BLUE, MPA: CYAN, APAP: MAGENTA, LORAZEPAM: DARK GREEN and (c): AMT: RED, ITZ: BLUE, DHVCZ: PURPLE, HVCZ: GOLD, 4HVCZ: OLIVE
