# Supplementary material for: A potential implication of UDP-glucuronosyltransferase 2B10 in the detoxification of drugs used in pediatric hematopoietic stem cell transplantation setting: an in silico investigation
Source: BMC Mol Cell Biol. 2022 Jan 21;23:5. doi: 10.1186/s12860-021-00402-5 (PMC8781437; doi:10.1186/s12860-021-00402-5)
Supplement: Supplementary file 10 — Additional file 10. Workflow of molecular docking predictions and molecular dynamics simulations performed to find potential UGT2B10 ligands. [file 12860_2021_402_MOESM10_ESM.docx]

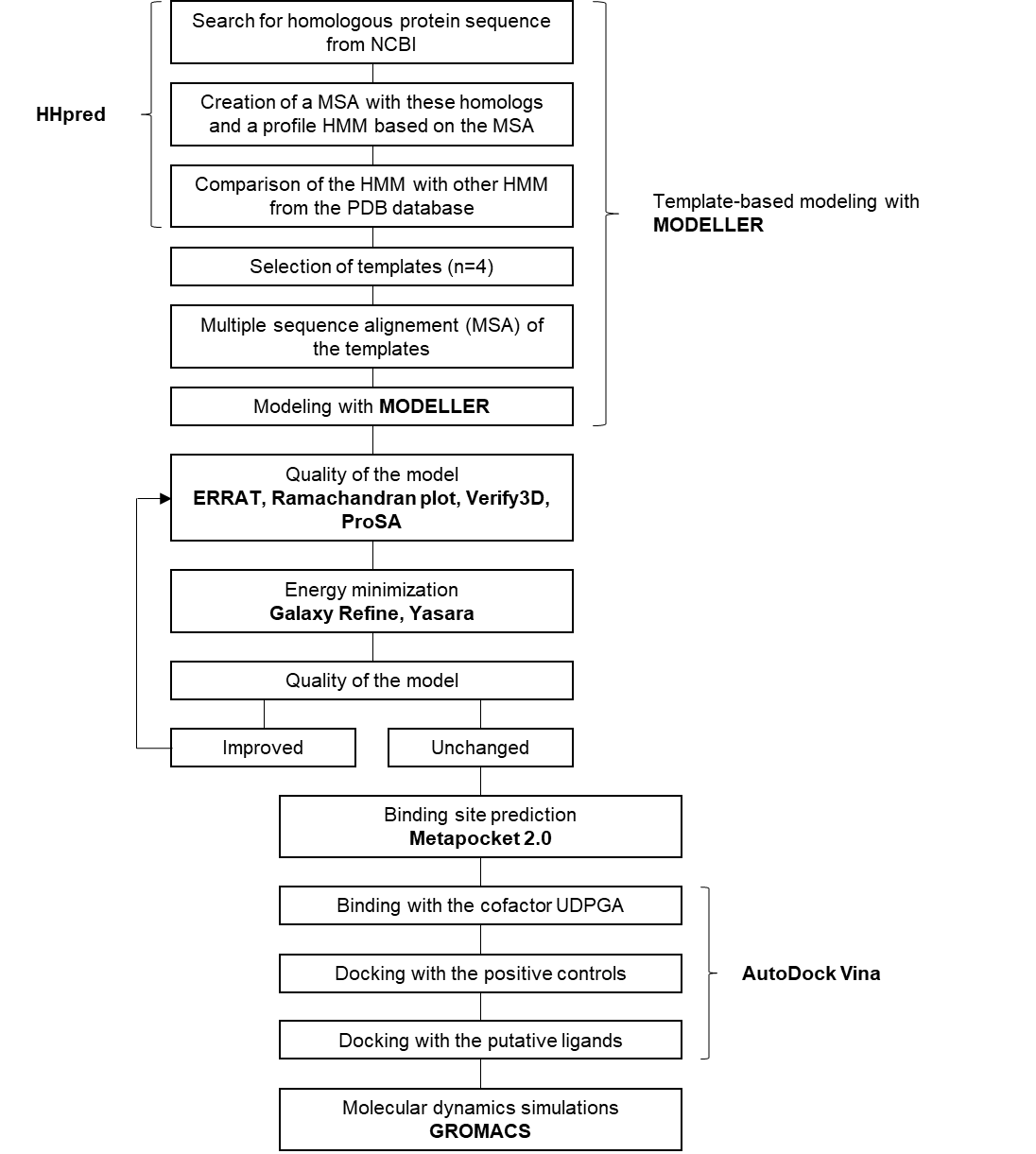


Additional file 10. Workflow of molecular docking predictions and molecular dynamics simulations performed to find potential UGT2B10 ligands.
