# Supplementary material for: A potential implication of UDP-glucuronosyltransferase 2B10 in the detoxification of drugs used in pediatric hematopoietic stem cell transplantation setting: an in silico investigation
Source: BMC Mol Cell Biol. 2022 Jan 21;23:5. doi: 10.1186/s12860-021-00402-5 (PMC8781437; doi:10.1186/s12860-021-00402-5)
Supplement: Supplementary file 11 — Additional file 11. List of tools, software and websites used for multiple template homology modeling of human UGT2B10, docking predictions and MD simulations. [file 12860_2021_402_MOESM11_ESM.docx]

| **Software/Tool/Website** | **Version** | **Usage** | **Source** |
| --- | --- | --- | --- |
| Uniprot | N/A | Retrieving protein sequence | ^6^ |
| HHpred | N/A | Search homologous protein with comparison of hidden Markov-Models profiles | ^7^ |
| InterPro | N/A | Classification of protein families and identification of important domains and conserved sites among these families. | ^8^ |
| MODELLER | 9.24 | Production of the homology modeling using selected protein structures as templates. Used as a module in the Chimera software | ^9^ |
| Chimera | 1.15 | Used to build the homology model using the MODELLER module and visualization of the results | ^10^ |
| PLIP | N/A | Overview of the structure and the important interacting residues in the protein | ^11^ |
| Yasara Minimization server | N/A | Energy minimization of the UGT2B10 model using the YASARA force field | ^12^ |
| GalaxyRefine | N/A | Energy minimization of the UGT2B10 model using repeated steps of perturbation and relaxation with molecular dynamics. | ^13^ |
| Verify3D | N/A | Determination of the protein 3D structure quality, based on the sequence. | ^14,15^ |
| ERRAT | N/A | Determination of the model quality, using the analysis of the nonbonded interaction between specific atom types | ^16^ |
| Structure Analysis and Verification (SAVES) servers | 6.0 | Used to build Ramachandran plot, to assess the quality of the model. | ^17^ |
| ProSA | N/A | Used to assess the quality of the model. This tool compares the results to experimentally determined protein chains, present in the PDB database | ^18^ |
| MetaPocket | 2.0 | Determination of the binding pockets of the model for docking predictions. The tool is based on a consensus from 6 different tools; LIGSITECS, PASS, Q-SiteFinder, SURFNET, Fpocket, GHECOM, ConCavity and POCASA | ^19^ |
| PyMol | 2.4.1 | Visualization of the docking results and preparation of the MD movie | ^20^ |
| QTGrace | 0.2.6 | Presentation of the MD simulation results analysis | ^21^ |
| PyRX | 0.8 | Used to screen the selected list of putative UGT2B10 ligands for their affinity towards UGT2B10 | ^22^ |
| AutoDock Vina | 1.1.2 | Used or molecular docking predictions between UGT2B10 and the list of putative ligands. Used as a module in PyRX | ^23^ |
| Corina Classic | 4.3.0 | Preparation of the ligand structure from SMILES to PDB format for docking | ^24^ |
| MGL Tools | 1.5.7 | Preparation of the UGT2B10 model and the ligand in PDBQT format | ^25^ |
| GROMACS | 5.1.4 | Molecular Dynamic simulations | ^26^ |

Additional file 11. List of tools, software and websites used for multiple template homology modeling of human UGT2B10, docking predictions and MD simulations.
